# A nuclear magnetic resonance based approach to accurate functional annotation of putative enzymes in the methanogen *Methanosarcina acetivorans*

**DOI:** 10.1186/1471-2164-12-S1-S7

**Published:** 2011-06-15

**Authors:** Yihong Chen, Ethel Apolinario, Libuse Brachova, Zvi Kelman, Zhuo Li, Basil J Nikolau, Lucas Showman, Kevin Sowers, John Orban

**Affiliations:** 1 Institute for Bioscience and Biotechnology Research, University of Maryland, 9600 Gudelsky Drive, Rockville MD 20850, USA; 2 Department of Marine Biotechnology, University of Maryland Baltimore County, Baltimore MD, USA; 3 Department of Biochemistry, Biophysics and Molecular Biology, Iowa State University, Ames IA 50011, USA; 4 Department of Cell Biology and Molecular Genetics, University of Maryland College Park, USA; 5 Center for Biorenewable Chemicals, Iowa State University, Ames IA 50011, USA; 6 Plant Sciences Institute, Iowa State University, Ames IA 50011, USA; 7 Department of Chemistry and Biochemistry, University of Maryland College Park, USA

## Abstract

**Background:**

Correct annotation of function is essential if one is to take full advantage of the vast amounts of genomic sequence data. The accuracy of sequence-based functional annotations is often variable, particularly if the sequence homology to a known function is low. Indeed recent work has shown that even proteins with very high sequence identity can have different folds and functions, and therefore caution is needed in assigning functions by sequence homology in the absence of experimental validation. Experimental methods are therefore needed to efficiently evaluate annotations in a way that complements current high throughput technologies. Here, we describe the use of nuclear magnetic resonance (NMR)-based ligand screening as a tool for testing functional assignments of putative enzymes that may be of variable reliability.

**Results:**

The target genes for this study are putative enzymes from the methanogenic archaeon *Methanosarcina acetivorans* (MA) that have been selected after manual genome re-annotation and demonstrate detectable *in vivo* expression at the level of the transcriptome. The experimental approach begins with heterologous *E. coli* expression and purification of individual MA gene products. An NMR-based ligand screen of the purified protein then identifies possible substrates or products from a library of candidate compounds chosen from the putative pathway and other related pathways. These data are used to determine if the current sequence-based annotation is likely to be correct. For a number of case studies, additional experiments (such as *in vivo* genetic complementation) were performed to determine function so that the reliability of the NMR screen could be independently assessed.

**Conclusions:**

In all examples studied, the NMR screen was indicative of whether the functional annotation was correct. Thus, the case studies described demonstrate that NMR-based ligand screening is an effective and rapid tool for confirming or negating the annotated gene function of putative enzymes. In particular, no protein-specific assay needs to be developed, which makes the approach broadly applicable for validating putative functions using an automated pipeline strategy.

## Background

Protein functions are annotated in genomic databases using automated routines that search for sequence homology to a gene product with an established function. The accuracy of these sequence-based annotations is often variable, particularly if the sequence identity to a known function is low. Indeed recent work has shown that even proteins with very high sequence identity can have different folds and functions [1-3], and therefore caution is needed in assigning functions simply by sequence homology in the absence of experimental validation. Traditional experimental approaches to determine function such as enzyme assays are slow and painstaking and have not been able to keep up with the ever-increasing large body of genome sequence data that contains many genes with unconfirmed and undetermined function. Clearly more efficient methods for accurate, experimental-based annotation and validation of function are needed.

One area where there is a strong demand for functional annotation is the large number of putative enzymes identified from structural genomics and other efforts (e.g. [4-7]). Methods for rapidly establishing small molecule substrate or product specificity of putative enzymes are likely to be extremely useful on two levels. Firstly, they would allow efficient testing of functional assignments that may be of variable reliability. Secondly, such approaches may be extended to the characterization of partially assigned enzymatic functions like those annotated from structural genomics efforts. This article discusses a method that is applicable to the first level of testing current sequence-based annotations of enzymatic function. An NMR-based approach is described for identifying potential substrates or products of enzymes *in vitro*.

For the goal here of developing rapid approaches for annotating putative enzymes, we adopted a ligand-based NMR screening strategy [8]. In our hands, the most consistent results were obtained using the waterLOGSY (water-ligand observed *via* gradient spectroscopy) pulse sequence [9]. This method was originally developed for ligand screening of drug targets and is amenable to a pipeline approach. The NMR experiment is based on magnetization transfer between ligand and water molecules. In the presence of a protein that binds to the ligand, there are two competing flows of magnetization: 1) from water to the free ligand and 2) from bound water (*via* the protein) to the bound ligand. These two flows lead to opposite signs of the NOEs (nuclear Overhauser enhancements) between water and the ligand. The stronger magnetization flow determines the sign of the waterLOGSY peak. Compounds that bind the protein will give positive peaks whereas peaks generated from non-binding compounds will be negative in the waterLOGSY spectrum. Since exchangeable protons (e.g. hydroxyl or amino group protons) also appear as positive peaks in waterLOGSY spectra, these need to be identified and deconvoluted from the peaks due to protein-binding. This is readily achieved by recording a reference spectrum of the sample in which the water signal is saturated. Through chemical exchange, the labile OH and NH protons are also saturated and their peak intensities are greatly decreased allowing straightforward distinction of peaks due to binding.

The case studies below illustrate how this method can be used to identify the chemical structures of potential substrates or products for putative enzyme proteins. The functional assignments were further supported by additional experiments (e.g. genetic complementation, NMR-based enzyme assays). In all of the examples studied, we find that the initial NMR screen is indicative of whether the functional annotation is correct.

## Results and discussion

### Choice of organism and target selection

Genes from the metabolically diverse methanogenic archaeon, *Methanosarcina acetivorans*, were chosen for this study [10-12]. Methane producing organisms are of interest because they provide an efficient and cost-effective biofuel which is self-harvesting and can be distributed readily using existing infrastructure. As with other genomes, however, accurate functional annotation of methanogens lags significantly behind the large body of sequence data, representing a sizable gap in understanding of the biology of these organisms. This project was initiated by updating functional annotations for over 700 of the 4721 predicted genes in the MA genome. This was done by transferring many of the recently revised manual annotations in the closely related species *M. burtonii*[13] to homologous genes in MA. In combination, a thorough literature search was conducted for published data that experimentally confirms the functionality of MA genes and closely related orthologs in other species. A complete list of revised MA annotations is provided in Additional file 1 (also available at http://ibbr.umd.edu/g2f) with summary statistics in Additional file 2.

By analogy with the *M. burtonii* re-annotation, confidence levels were given to each re-annotation based on current literature as follows: *Level 1*: An exact match in the literature with an experimentally defined function. *Level 2*: Gene product contains all domains needed for enzymatic function with ≥35% sequence identity to a gene product of known function. *Level 3*: Gene product contains all domains needed for enzymatic function but ≤35% sequence identity to a gene product of known function. *Level 4*: Gene product has no experimental match but some domain similarities to a known function are recognizable. *Level 5*: Has no experimental match or domain similarities – i.e. annotated as hypothetical. This provided a list from which targets with varying confidence levels were selected for experimental validation using our pipeline approach.

The two main selection criteria were 1) the protein should have a putative enzymatic activity on a small molecule substrate and 2) the protein should be non-membranous based on amino acid sequence analysis. Additional characteristics that were preferable but not absolutely required were that the gene product was expressed *in vivo* in MA based on published reports [14] and that an *E. coli* homolog exists for potential genetic complementation studies. A total of 44 MA targets were cloned of which 27 were found to express soluble protein in *E. coli*. We describe here a number of these as case studies to illustrate our generalized approach.

### Case study #1: MA4265

**Figure 1:**
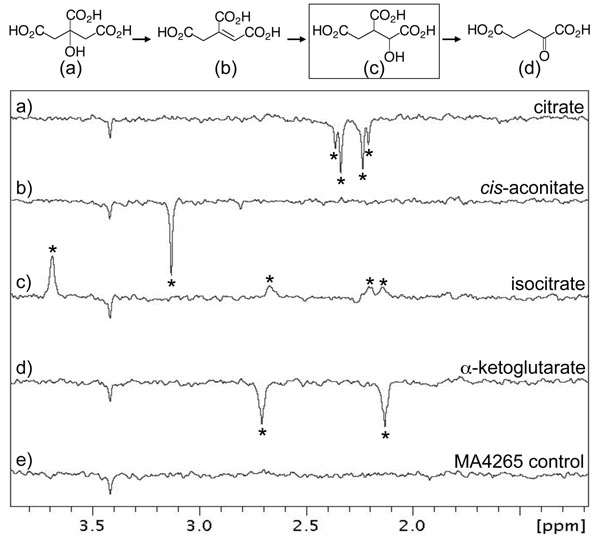
**NMR-based ligand screening of MA4265, a putative isocitrate/isopropylmalate dehydrogenase. **Part of the metabolic pathway involving isocitrate dehydrogenase functionality is shown at the top of the figure. The relevant section of the one-dimensional waterLOGSY ^1^H NMR spectrum is shown for each metabolite (300 μM) in the presence of MA4265 (30 μM) as follows: (a) citrate; (b) *cis*-aconitate; (c) isocitrate; (d) 2-ketoglutarate; (e) control spectrum of MA4265 alone. Peaks due to each compound are labeled with asterisks. Metabolite interaction with MA4265 is represented as positive peaks whereas negative peaks indicate no binding to the protein. The negative peak at 3.4 ppm in all spectra is thought to be a contaminant from the protein concentration process. Only isocitrate binds to MA4265 under the conditions used.

The MA4265 gene is annotated as a putative “isocitrate/isopropylmalate dehydrogenase family protein” in the DOE Integrated Microbial Genome (IMG) database (http://img.jgi.doe.gov/). The re-annotation to a putative isocitrate/isopropylmalate dehydrogenase, transferred from the function re-assignment of the *M. burtonii* homolog [13], is based on ~30-35% sequence identity to homologs where the function has been verified experimentally [15,16]. In our studies, the gene product of MA4265 (342 aa) was heterologously expressed in *E. coli* as a soluble protein at 25°C and was folded as judged by 1D ^1^H NMR spectroscopy. The putative enzymatic annotations, isocitrate dehydrogenase and/or isopropylmalate dehydrogenase, placed this gene product in the tricarboxylic acid (TCA) cycle and/or the leucine biosynthesis pathway, respectively. These predicted functional annotations were initially tested by waterLOGSY NMR screening with relevant intermediates of the TCA cycle and leucine biosynthesis pathways. NMR screening indicates that MA4265 interacts with isocitrate but not other intermediates in the TCA pathway (Figure 1) while no interactions were detected with intermediates of the leucine metabolic pathway (data not shown). The NMR data therefore provides a rapid *in vitro* screen supporting the assignment of the isocitrate dehydrogenase function to MA4265.

**Figure 2:**
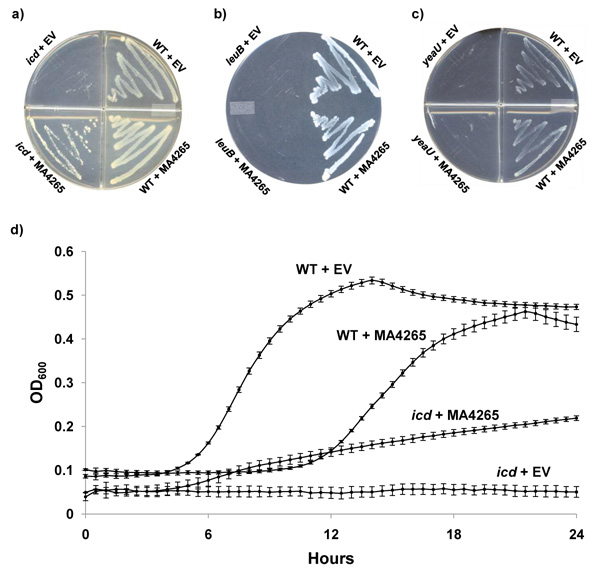
**Genetic complementation results for MA4265.** Growth of isogenic *E. coli* strains in the BW25113 (WT) background carrying deletion mutant alleles *icd* (a), *leuB* (b), and *yeaU* (c), and transformed with an empty control vector (EV=pET-21a) or a plasmid expressing MA4265. All strains were grown on M9 minimal media with glucose (or malate in plate c) as the carbon source, and contained 100 µg/mL ampicillin. Plates were incubated at 37°C for 48 hr. For panel (c), D-malate was substituted for glucose as the sole carbon source to examine the growth phenotype of *yeaU*[23]. d) The *icd* mutant strain carrying either the empty control vector (*icd*+EV) or the MA4265-expressing vector (*icd*+MA4265) and the parental strain (WT) empty control vector (WT+EV) or the MA4265-expressing vector (WT+MA4265) were grown in M9 liquid media with glucose as the carbon source and 100 µg/mL ampicillin. Cultures were incubated at 37°C and OD_600_ was monitored. Data from the average of three replicates (± standard error) are presented.

The functional annotation of MA4265 was further tested using genetic complementation studies. The *E. coli* genome contains three homologs of MA4265: *yeaU*, a putative tartrate dehydrogenase (Bit score 164, E 1e-41, 34% seq id); *leuB*, 3-isopropylmalate dehydrogenase (Bit score 146, E 2e-36, 32% seq id); and *icd*, isocitrate dehydrogenase (Bit score 130, E 1e-31, <32% seq id). For each of these homologs, a gene knockout mutant strain is available in the Keio collection [17] for complementation studies. *E. coli* mutant strains carrying knockout alleles in these three homologs show lethal growth phenotypes when grown on minimal salt media with glucose as the carbon source. Figure 2a shows that the lethal growth phenotype of *icd* can be partially recovered by expressing MA4265, while the expression of this gene was insufficient to rescue the growth of the *leuB* and *yeaU* mutants (Figures 2b and 2c). To quantify the capacity of MA4265 to complement the *icd* mutation, growth in liquid medium was monitored (Figure 2d). In liquid M9 minimal media with a glucose-carbon source, no growth was observed in the *icd*-mutant strain transformed with an empty vector. In contrast, the *icd* mutants transformed with the plasmid expressing MA4265 displayed growth with a doubling time of 4.37±0.09 hours, which is slightly slower than the doubling time of 3.85±0.10 hours for the parental control *E. coli* strain (BW25113) expressing the same MA4265 construct. In both BW25113 and the *icd* mutant, expression of MA4265 resulted in doubling times that were moderately slower than BW25113 containing an empty pET-21a vector with a doubling time of 1.96±0.02 hours.

Thus the NMR and gene complementation data are both consistent with the isocitrate dehydrogenase function, but do not support the 3-isopropylmalate dehydrogenase or tartrate dehydrogenase annotations.

### Case study #2: MA0940

**Figure 3:**
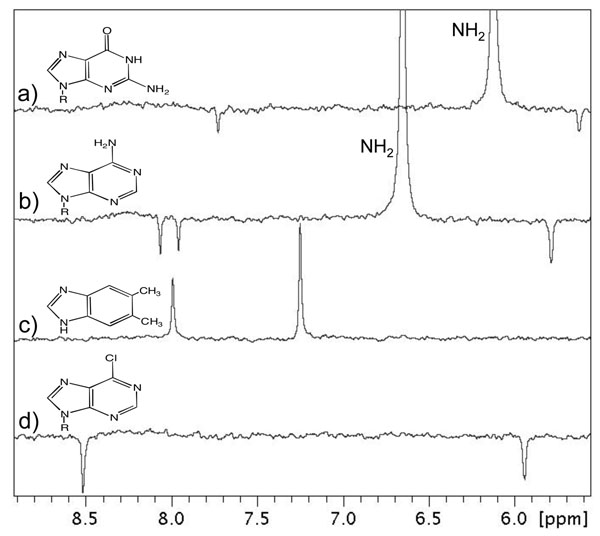
**Ligand screening of selected purine-type nucleobase derivatives against the MA0940 gene product. **Compounds screened are (a) guanosine, (b) adenosine, (c) 5,6-dimethylbenzimidazole, and (d) 6-chloropurine riboside. The peaks labeled NH_2_ in (a) and (b) are exchangeable purine amino groups that are manifested as positively phased peaks in the waterLOGSY experiment.

MA0940 is currently annotated as a hypothetical protein in the IMG database. Our revised annotation of MA0940 as encoding alpha-ribazole-5’-phosphate phosphatase (CobZ) is based on a literature search which identified an experimental study of a *Methanosarcina mazei* homolog that is 91% identical to MA0940 [18]. Because there are known instances in which proteins with high sequence identity can adopt alternative folds and functions [1-3], a rapid experimental test is needed that can verify or increase confidence in the annotation of MA0940 as encoding a CobZ ortholog. In this example the putative substrate and product were not commercially available but the use of suitable structural or sub-structural analogs in NMR screening provided insights into substrate recognition by MA0940 [6]. NMR-based ligand screening of the nucleobase substructure of alpha-ribazole, 5,6-dimethylbenzimidazole, which is commercially available, indicated that this compound does interact with MA0940. In contrast, similar compounds containing bicyclic aromatic rings such as adenine and guanine derivatives do not bind to MA0940, indicating that this gene product has a binding preference for the dimethylbenzimidazole moiety (Figure 3) and supporting the assignment of its function as alpha-ribazole-5’-phosphate phosphatase. The earlier study on the *M. mazei* homolog, Mm2058, was done using gene complementation experiments which can be quite time consuming. The revised MA0940 functional assignment, while not as definitive as the *M. mazei* study, is supported through a rapid *in vitro* screening process that does not require any specialized reagents (substrates, products, organism-specific gene knockouts) or pathway-specific knowledge.

### Case study #3: MA3706

MA3706 is annotated as a putative Ham1 protein in the IMG database and our re-annotation process does not change this annotation. Ham1 proteins are nucleoside triphosphatases that are hypothesized to catalyze the hydrolysis of non-standard nucleoside triphosphates (NTPs) to nucleoside monophosphates as a mechanism for preventing their incorporation into DNA and RNA [19]. In particular, they are thought to target the oxidatively modified inosine and xanthosine triphosphates. Our annotation of MA3706 is based on homology with Mj0226 from *Methanococcus jannaschii*[20,21]. MA3706 and Mj0226 share 47% sequence identity and the latter has been shown to preferentially hydrolyze xanthosine triphosphate (XTP) and deoxyinosine triphosphate (dITP) over other canonical nucleoside triphosphates. We therefore tested whether MA3706 interacts with nucleotides in a similar way by screening a series of standard and modified NTPs from our small molecule library for binding with MA3706.

**Figure 4:**
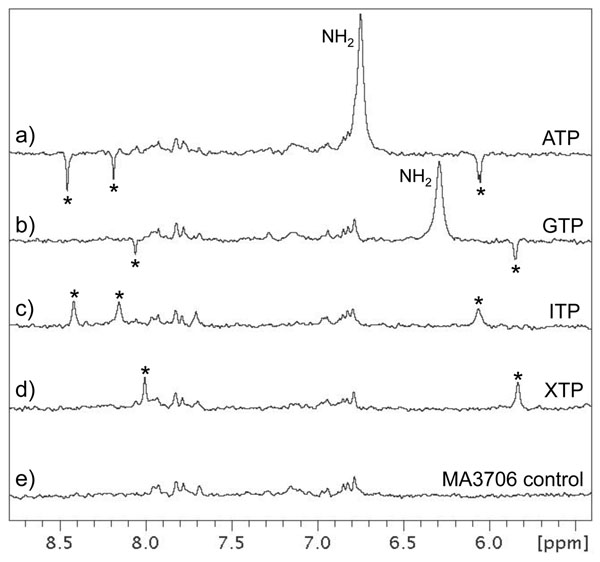
**NMR waterLOGSY spectra for a series of standard and modified nucleotide triphosphates with MA3706 protein. **Nucleotides screened are (a) adenosine triphosphate, (b) guanosine triphosphate, (c) inosine triphosphate, and (d) xanthosine triphosphate. A control spectrum of MA3706 is shown in (e). Peaks due to nucleotide protons are labeled with an asterisk in each spectrum. The exchangeable amino groups of ATP and GTP are labeled in (a) and (b).

**Figure 5:**
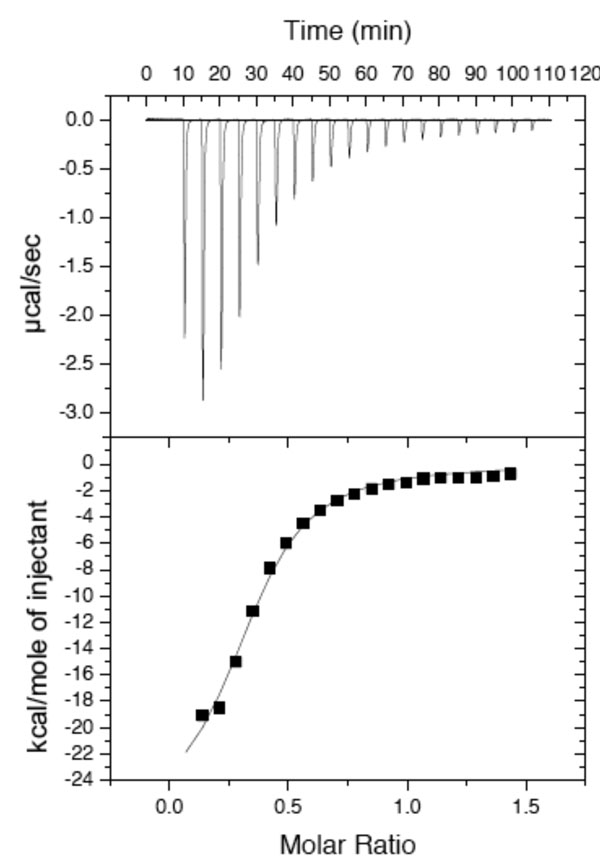
Isothermal titration calorimetric data for the binding interaction between ITP and MA3706.

**Figure 6:**
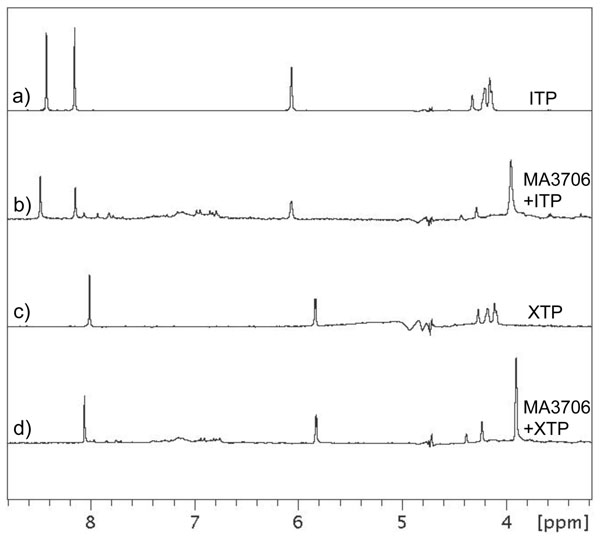
**Conventional 1D ^1^H NMR spectra showing enzymatic activity of MA3706 in the presence of magnesium chloride.** (a) ITP alone at 500 μM concentration; (b) 500 μM ITP mixed with 50 μM MA3706 in the presence of 1 mM magnesium chloride; (c) XTP alone at 500 μM concentration; (d) 500 μM XTP mixed with 50 μM MA3706 in the presence of 1 mM magnesium chloride.

Standard NTPs do not bind to MA3706 based on the negative peaks due to nucleotide observed in waterLOGSY spectra (e.g. ATP and GTP, Figures 4a and 4b). However, positive peaks indicative of a binding interaction are detected when MA3706 is mixed with the non-canonical nucleotides ITP (Figure 4c) and XTP (Figure 4d). Binding of MA3706 to ITP was also characterized using isothermal titration calorimetry and a dissociation constant of 7.8 μM was obtained for this interaction (Figure 5). Because divalent cations are known to be essential for the enzymatic activity of many hydrolytic enzymes, magnesium chloride was added to the NMR samples to see if any chemical change in the nucleotides could be detected using 1D ^1^H NMR spectroscopy. Indeed, the NMR spectra of both free ITP and XTP differed substantially from their spectra in the presence of MA3706 and magnesium chloride (Figure 6). The largest changes occurred in the ribose proton region between 3.8-4.5 ppm, indicating that ITP and XTP had been chemically transformed. Comparison with NMR spectra of reference compounds (data not shown) showed that the new species generated in this reaction matched exactly with the corresponding nucleoside monophosphates IMP and XMP. No signals due to any residual nucleoside triphosphates or diphosphates were evident. Control experiments with the standard nucleotides ATP and GTP did not show any changes in their NMR spectra in the presence of MA3706 and magnesium ions. These results indicate unambiguously that MA3706 has both an inosine triphosphate pyrophosphatase and xanthosine triphosphate pyrophosphatase biochemical function, consistent with the annotation of Mj0226.

Thus the NMR ligand screening approach provides a very efficient means for identifying the nucleotide binding preferences of MA3706. Further, once binding specificity was established, the enzymatic activity was detected directly in the NMR sample without the need for involved assays.

### Other examples

Using the approach described, a number of other MA gene product annotations were also investigated. Table 1 summarizes the genes that were studied. The experimental data can be put into 3 categories. In several examples (MA0940, MA2498, MA3520, MA3706) the data are consistent with the putative biochemical function and therefore provide increased confidence in the existing annotation. This sometimes occurs even when the sequence homology to an ortholog of known function is not very high (e.g. MA2498). Other examples such as MA4265 show that the existing annotations are only partially correct. Here, the experimental data suggest that the function assignment needs to be narrowed. A third category contains genes where the present functional assignment is not supported by our experimental screening procedure. For example MA0154 is currently annotated as biotin synthase in the IMG database, but our NMR-based ligand screening of the gene product did not detect binding to the putative substrate, product, or any other compounds in the biotin pathway (data not shown). A report published subsequent to our testing showed that this gene is in fact involved in pyrrolysine biosynthesis [22]. This highlights another general problem with regard to assignment of function where database entries are sometimes not updated after the initial annotation. Nevertheless, NMR screening was quickly able to detect that the ligand binding results were not consistent with the IMG annotation, indicating that this function assignment was likely to be incorrect.

## Conclusions

Correct annotation of function is essential if one is to take full advantage of the vast amounts of genomic sequence data. Incorrect assignment of function is propagated by comparative annotation with mis-annotated genes and can potentially lead to mis-placed experimental efforts. Conversely, a corrected annotation in one organism can provide tremendous leverage in re-annotation of orthologs from a diverse phylogeny of organisms. Experimental methods are therefore needed to efficiently evaluate annotations in a way that complements current high throughput sequence homology-based techniques.

We describe here an NMR-based approach for rapidly and reliably testing functional assignments of putative enzymes. Most importantly, no protein-specific assay that involves a chemical conversion needs to be developed for the initial screening, which makes the approach broadly applicable for validating putative functions using an automated pipeline strategy. Thus the ligand screen provides guidance about which small molecules may serve as suitable substrates for an enzyme assay that can be developed subsequently. The case studies described here, as well as other examples summarized in Table 1, demonstrate that the NMR screen provides a quick indication of whether the putative function assignment is likely to be correct. This is done by identifying small molecule ligands that can act as either substrates or products, or their structural analogs. Notably it was even possible to directly detect an enzymatic activity in the NMR tube for some examples (MA3706, MA2498) when the necessary cofactors are present.

**Table 1:** Summary of MA genes studied

MA Gene	IMG Annotation^1^	Experimental data consistent with IMG annotation?	Comment^2^
MA0154 (350 aa)	Biotin synthase	No	Revised annotation is to pyrrolysine biosynthesis protein
MA0246 (422 aa)	4-Hydroxybenzoate decarboxylase	No	Revised annotation is 3-octaprenyl-4-hydroxybenzoate carboxylyase
MA0940 (183 aa)	Conserved hypothetical	-	Revised annotation is alpha-ribazole-5’-phosphate phosphatase and experimental data is consistent with this
MA2498 (196 aa)	Fumarate hydratase	Yes	Revised annotation is fumarate hydratase/tartrate dehydratase but experimental data is only consistent with the former
MA3520 (412 aa)	Glycine hydroxymethyl-transferase	Yes	No change in annotation
MA3706 (184 aa)	Ham1 protein/nucleotide triphosphatase	Yes	No change in annotation
MA4265 (342 aa)	Isocitrate/ isopropylmalate dehydrogenase family protein	Partially	Revised annotation is isocitrate/ isopropylmalate dehydrogenase but the experimental data is only consistent with the former

^1^ Obtained from the DOE Integrated Microbial Genomes database (http://img.jgi.doe.gov/); ^2^ Revised annotations were based on comparison with the updated annotations in *M. burtonii*[13] as described in the text.

For the examples described here, where there is some pre-existing annotation of putative function, the ligand screening is targeted and generally involves fewer than 20 compounds per protein. Where even less is known about gene function (e.g. a “putative methyltransferase” annotation), a larger number of compounds will need to be screened. However, it is possible to develop a suite of compounds for screening in an automated fashion. We use a 24-sample robot for most screening applications, with automated sample change, shimming, acquisition and processing. Typically we use 1-5 compounds per protein sample depending on how many compounds need to be screened. Pooled compounds need to have at least one resolvable ^1^H NMR signal and be structurally as diverse as possible to minimize the chance of competition for binding. One limitation of this approach is that the most relevant compounds for testing may sometimes not be commercially available. Nevertheless, as demonstrated in this report, structural analogs can often be used to gain insights into the types of small molecules recognized by the gene product even when the exact substrate or product is not readily available (e.g. MA0940).

In principle, the approach described here is applicable to putative enzymes with completely undefined substrate specificity. Further studies coupling NMR-screening with other methods such as mass spectrometry-based metabolite profiling will be needed to determine functions for the large numbers of such putative enzymes that are currently poorly defined.

## Methods

### Cloning of target genes

Target genes were PCR-amplified from isolated MA genomic DNA using primer sets listed in Additional file 3. Invitrogen’s Platinum^®^*Pfx* DNA Polymerase protocol was followed. Several of the PCR products were treated with Taq DNA polymerase (PE Applied Biosystems) and 2.5 μmol dATP (Roche) for cloning into the Invitrogen pCR4-TOPO vector. TOPO ligations were transformed into DH5α competent *E. coli* cells (Invitrogen), selected on LB-ampicillin, and sequenced. These clones were then used for subsequent cloning into the pET-21a vector (Novagen). For other clones, the PCR products were cloned directly into the pET vector and sequenced. All constructs contained a C- or N-terminal His_6_-tag, introduced via the PCR primers.

### Protein expression and purification

MA proteins were over-expressed in *E. coli* BL21(DE3) Rosetta cells (Invitrogen) transformed with the plasmid constructs containing the target MA genes. Optimal temperatures for expression were determined using small-scale (10 mL) trial LB cultures at 16°C, 25°C or 37°C in the presence of antibiotic. If soluble expression could not be obtained at any of these temperatures, expression at 10-13°C with ArcticExpress cells (Stratagene) for 24 hours was also attempted. In a number of cases this produced soluble protein.

For NMR studies, a 1 L culture was incubated at 37°C until the OD_600_ reached 0.4-0.8. The temperature was then adjusted to the pre-determined optimum and expression was induced with 1 mM IPTG. Typical expression times ranged from 5-24 hours. Cells were harvested by centrifugation (3500g, 30 min) and the pellet was re-suspended in binding buffer (10 mM imidazole, 300 mM sodium chloride, 50 mM sodium phosphate, pH 8.0). The cells were then lysed by sonication and centrifuged (35000g, 1 hr). The supernatant was loaded on a Ni-NTA-Agarose column (Qiagen) and purified with an imidazole gradient using standard procedures. Pure fractions were combined and dialysed against a standard buffer for NMR samples (50 mM sodium phosphate, 100 mM sodium chloride, pH 7.0).

### NMR-based ligand screening

NMR experiments were acquired at 5°C and 25°C on a Bruker DMX-600 spectrometer equipped with either a Z-axis gradient cryoprobe or a conventional 3-axis gradient probe. The typical protein concentration used for NMR experiments was in the 10-50 μM range. Initial test compound concentrations were set at ten times the protein concentration. This allowed detection of binding in the 0.1 micromolar to hundreds of micromolar range. If higher compound to protein ratios (e.g. 100:1) are used then only the tightest micromolar binders are detected. Thus the stringency of the experiment can be controlled by the compound-to-protein ratio. Test compounds were generally prepared as 50 or 100 mM stock solutions in *d6*-DMSO or water and diluted appropriately into NMR samples. Compounds were obtained from Sigma-Aldrich.

One-dimensional ^1^H NMR waterLOGSY experiments were acquired using established protocols [9]. A reference experiment was collected followed by the waterLOGSY magnetization transfer spectrum. Typical acquisition parameters for the waterLOGSY spectra were 256-512 transients with a mixing time of 1.5 s and a 2 s relaxation delay. Using these parameters each experiment took 15-30 minutes to acquire. NMR spectra were processed using Bruker Topspin software (version 1.3) and analyzed by electronically overlaying reference and waterLOGSY spectra in dual display mode. A Bruker NMRCase sample changer robot controlled by ICON-NMR software was used for automated sample change, shimming, data acquisition (reference and waterLOGSY), and processing.

### Gene complementation

Genetic complementation of *E. coli* mutants by the MA genes was performed with *E. coli* deletion mutants generated in the BW25113 background [17]. The specific mutant strains used in this study were: JW1122 (*icd*), JW5807 (*leuB*), and JW1789 (*yeaU*). All mutant strains and the isogenic wild-type control strain were lysogenized with lambda-DE3 (lambda-DE3 lysogenization kit; Novagen). Each strain was transformed with a non-recombinant control pET-21a plasmid (Novagen) and a recombinant pET-21a plasmid carrying a cloned MA gene. Transformants were selected by the ability to grow on a medium containing 100 µg/mL ampicillin, and this medium also contained 50 µg/mL kanamycin, for maintaining selection of the deletion mutant allele.

Complementation was demonstrated on 1.5% agar plates by streaking selected transformants from LB medium to M9 minimal agar media containing 100 µg/mL ampicillin in the presence or absence of 0.1 mM IPTG, and grown at 37°C. Glucose was used as the carbon source with the exception of the experiment shown in Figure 2c where the wild type control and *yeaU* mutants were plated on 2g/L D-malate as the sole carbon source [23].

Growth curves were produced by growing strains in liquid cultures in 48-well culture plates that were incubated at a constant temperature of 37°C. The medium consisted of M9 minimal media with glucose as the carbon source, containing 100 µg/mL ampicillin in the presence or absence of 0.1 mM IPTG. The liquid cultures were inoculated to an OD_600_ of ~0.05 from an inoculum culture grown overnight in LB with the appropriate antibiotics. Cells were collected by centrifugation, washed, and re-suspended with M9 media prior to inoculation. A Multi-Detection Microplate Reader Synergy HT (BioTech) was used to simultaneously measure the OD_600_ at 30-minute intervals.

### Isothermal titration calorimetry

The binding interaction between ITP and MA3706 was quantified using a Microcal VP Titration Calorimeter. The protein was dialyzed into buffer containing 50 mM sodium chloride, 100 mM sodium phosphate (pH 7.0). ITP (Sigma) was dissolved in the protein dialysis buffer at a concentration of 1.0 mM. Five microliters of ITP were injected into a 50 μM solution of MA3706 every 5 min until MA3706 was saturated.

## Author contributions

JO set up NMR experiments and the small molecule library, interpreted NMR and ITC data, participated in the manual re-annotation, and drafted the manuscript. YC expressed and purified proteins, helped to acquire NMR data, and performed the calorimetry experiments. EA cloned MA genes and participated in the manual re-annotation. LB assisted in setting up the genetic complementation experiments. ZK designed strategies for cloning MA genes, assisted in the manual re-annotation, and participated in drafting the manuscript. ZL cloned MA genes and assisted in the manual re-annotation. BJN supervised the genetic complementation experiments and participated in drafting the manuscript. LS performed the genetic complementation study. KS coordinated cloning and manual re-annotation, and participated in drafting the manuscript.

## Competing interests

The authors declare that they have no competing interests.

## Supplementary Material

Additional file 1 **List of revised MA annotations** Annotations for genes between MA0001 and MA4675. Click here for file

Additional file 2 **Summary of results for manually re-annotated MA genes.** (a) Categorization of revised MA annotations as more specific, less specific or no change. (b) The distribution of confidence levels (defined in the text) in re-annotated MA genes. Click here for file

Additional file 3 **List of forward and reverse primer** DNA sequences used for cloning MA target genes. Click here for file
