# Supplementary material for: A nuclear magnetic resonance based approach to accurate functional annotation of putative enzymes in the methanogen Methanosarcina acetivorans
Source: BMC Genomics. 2011 Jun 15;12(Suppl 1):S7. doi: 10.1186/1471-2164-12-S1-S7 (PMC3223730; doi:10.1186/1471-2164-12-S1-S7)
Supplement: Additional file 1 — List of revised MA annotations Annotations for genes between MA0001 and MA4675. [file 1471-2164-12-S1-S7-S1.pdf]

Locus_tag	Length	Gi	Old Product Name	New Product Name (a)	Confidence Level	Expressed in MA (b)	Change in Annotation	COG(s)
MA0001	420	20088900	cell division control protein 6	ORC complex protein Cdc6/Orc1	CL2	nd	more specific	COG1474LO
MA0002	125	20088901	hypothetical protein MA0002	hypothetical protein MA0002	CL5	nd	no change	-
MA0003	514	20088902	sodium:proline symporter	sodium:proline symporter putP	CL2	nd	no change	COG0591ER
MA0004	257	20088903	IstB helper protein	IstB helper protein	CL2	nd	no change	COG1484L
MA0005	414	20088904	transposase	transposase	CL3	nd	no change	COG4584L
MA0006	395	20088905	L-tyrosine decarboxylase	L-tyrosine decarboxylase	CL2	Yes	no change	COG0076E
MA0010	297	20088909	tetrahydromethanopterin formyltransferase	formylmethanofuran--tetrahydromethanopterin formyltransferase (ftr) (EC 2.3.1.101)	CL2	Yes	more specific	COG2037C
MA0031	296	20088930	pyruvate synthase, subunit beta	Pyruvate synthase beta chain (pyruvate:ferredoxin oxidoreductase beta subunit) (EC 1.2.7.1)	CL2	Yes	no change	COG1013C
MA0032	403	20088931	pyruvate synthase, subunit alpha	Pyruvate synthase subunit porA (Pyruvate oxidoreductase alpha chain) (EC 1.2.7.1) (porA)	CL2	Yes	no change	COG0674C
MA0033	86	20088932	pyruvate synthase, subunit delta	Pyruvate synthase delta chain (pyruvate:ferredoxin oxidoreductase delta subunit) (EC 1.2.7.1)	CL2	Yes	no change	COG1144C
MA0034	182	20088933	pyruvate ferredoxin oxidoreductase subunit gamma	Pyruvate synthase gamma chain (pyruvate:ferredoxin oxidoreductase gamma subunit) (EC 1.2.7.1)	CL2	Yes	no change	COG1014C
MA0042	415	20088941	peptide chain release factor 1	peptide chain release factor eRF/aRF, subunit 1	CL2	Yes	no change	COG1503J
MA0055	56	20088954	hypothetical protein	hypothetical protein	CL5	nd	no change	COG3609K
MA0056	71	10000000	hypothetical protein	conserved hypothetical protein	CL5	Yes	no change	COG3269R
MA0057	71	4E+11	hypothetical protein	conserved hypothetical protein	CL5	Yes	no change	COG3269R
MA0058	71	1.00E-30	hypothetical protein	putative uncharacterized protein	CL5	Yes	no change	COG3269R
MA0059		9.00E-32	hypothetical protein	conserved hypothetical protein	CL5	Yes	no change	COG3269R
MA0063	268	20088962	sulfonate ABC transporter, ATP-binding protein	ABC transporter ATPase subunit (ssuB)	CL2	nd	less specific	COG1116P
MA0064	262	20088963	sulfonate ABC transporter, permease protein	ABC transporter permease protein	CL4	Yes	less specific	COG0600P
MA0065	337	20088964	sulfonate ABC transporter, solute-binding protein	ABC transporter, substrate binding protein	CL4	Yes	less specific	COG0715P

MA0067	173	20088966	hypothetical protein MA0067	Helix-turn-helix (HTH) 3-containing transcriptional regulator, XRE family	CL4	Yes	more specific	COG4800K
MA0068	1167	20088967	hypothetical protein MA0068	Protein of unknown function DUF1608	CL4	Yes	more specific	COG1472G
MA0072	794	20088971	anaerobic ribonucleoside-triphosphate reductase	anaerobic ribonucleoside-triphosphate reductase	CL3	Yes	no change	COG1328F
MA0074	267	20088973	DNA repair and recombination protein RadB	DNA repair and recombination protein RadB	CL2	Yes	no change	COG0468L
MA0076	348	20088975	translation initiation factor IF-2B subunit alpha	Ribose-1,5-bisphosphate isomerase	CL2	Yes	more specific	COG0182J
MA0082	69	20088981	hypothetical protein MA0082	Hypothetical protein	CL5	nd	no change	-
MA0086	552	20088985	Hsp60	Thermosome subunit (Chaperonin)	CL2	Yes	no change	COG0459O
MA0090	539	20088989	O-phosphoseryl-tRNA synthetase	O-phosphoseryl-tRNA(Cys) synthetase (EC 6.1.1.-)	CL2	Yes	no change	COG2024J
MA0092	97	20088991	hypothetical protein MA0092	Protein of unknown function UPF0147	CL4	Yes	more specific	COG1698S
MA0096	821	20088995	Hef nuclease	Hef helicase	CL2	nd	more specific	COG1111L, COG1948L
MA0097	612	20088996	glycyl-tRNA synthetase	Glycyl-tRNA synthetase (EC6.1.1.14)	CL3	Yes	no change	COG0423J
MA0104	267	20089003	stationary phase survival protein SurE	Stationary-phase survival protein SurE (5'/3'-nucleotidase (EC 3.1.3.5) (EC 3.1.3.6); Exopolyphosphatase (EC 3.6.1.11)) (surE)	CL3	Yes	no change	COG0496R
MA0110	245	20089009	DNA polymerase sliding clamp	proliferating cell nuclear antigen (PCNA)/archaeal DNA polymerase sliding clamp	CL2	Yes	more specific	COG0592L
MA0113	138	20089012	ADP-ribose pyrophosphatase	ADP-ribose pyrophosphatase (Nudix hydrolase)	CL2	nd	no change	COG1051F
MA0117	118	20089016	hypothetical protein MA0117	Protein of unknown function with DUF381and DUF372 domains	CL4	nd	more specific	COG2098S
MA0119	405	20089018	acetylornithine aminotransferase	Acetylornithine/acetyl-lysine aminotransferase (EC 2.6.1.11) (EC 2.6.1.-)	CL2	Yes	more specific	COG4992E
MA0130	346	20089029	phosphoribosylaminoimidazole synthetase	Phosphoribosylformylglycinamidine cyclo-ligase (AIRS) (purM) (EC 6.3.3.1)	CL2	Yes	no change	COG0150F
MA0136	150	20089035	dCMP deaminase	dCMP deaminase (EC 3.5.4.12)	CL2	nd	no change	COG2131F
MA0137	150	20089036	dCMP deaminase	dCMP deaminase (EC 3.5.4.12)	CL2	Yes	no change	COG2131F
MA0143	468	20089041	ethanolamine permease	amine permease	CL4	nd	less specific	COG0531E

MA0145	218	20089043	monomethylamine corrinoid protein (mtmC)	monomethylamine corrinoid protein (mtmC1)	CL2	Yes	no change	COG5012R
MA0150	540	20089048	methylamine methyltransferase corrinoid activation protein	methylamine methyltransferase corrinoid activation protein	CL1	Yes	more specific	COG1145C, COG3894R
MA0152	259	2.00E-07	hypothetical protein	pyrrolysine synthesis protein	CL1	Yes	more specific	
MA0153	363	2.00E-07	hypothetical protein	pyrrolysine synthesis protein	CL1	Yes	more specific	
MA0154	350	20000000	biotin synthase (bioB)	pyrrolysine synthesis protein	CL1	Yes	more specific	
MA0155	443	161484944	pyrolysyl-tRNA synthetase	Pyrrolysyl-tRNA synthetase (pylS) (EC 6.1.1.-)	CL1	Yes	no change	COG0072J, COG2024J
MA0169	450	20089067	class H tetracycline resistance efflux protein	major facilitator superfamily transporter, large fragment ; major facilitator superfamily transporter, small fragment	CL4	nd	more specific	COG0477GEPR
MA0171	492	20089069	phenylalanyl-tRNA synthetase subunit alpha (pheS)	Phenylalanyl-tRNA synthetase, alpha subunit (EC 6.1.1.20)	CL2	nd	no change	COG0016J
MA0172	437	20089070	tryptophanyl-tRNA synthetase (trpS)	Tryptophanyl-tRNA synthetase (EC 6.1.1.2)	CL3	Yes	no change	COG0180J
MA0182	202	20089080	translation initiation factor IF-2 subunit beta (eif5)	translation initiation factor a/eIF-2 beta subunit	CL2	Yes	no change	COG1601J, COG3269R
MA0183	173	20089081	50S ribosomal protein L10e	LSU ribosomal protein L10AE (rpl10e)	CL2	Yes	no change	COG0197J
MA0194	925	20089092	alanyl-tRNA synthetase	Alanyl-tRNA synthetase (alaS) (EC 6.1.1.7)	CL3	Yes	no change	COG0013J
MA0201	375	20089099	3-isopropylmalate dehydrogenase (leuB)	Tartrate dehydrogenase/decarboxylase (EC 1.1.1.93) (EC 4.1.1.73) (EC 1.1.1.83)	CL2	Yes	more specific	COG0473CE
MA0202	162	20089100	3-isopropylmalate dehydratase small subunit (leuD)	3-Isopropylmalate dehydratase small subunit (3-isopropylmalate isomerase small subunit) and homoaconitase small subunit - dual specificity	CL2	Yes	more specific	COG0066E
MA0206	368	20089104	5-aminoimidazole-4-carboxamide-1-beta-D-ribofuranosyl 5'-monophosphate--formate ligase	5-formaminoimidazole-4-carboxamide-1-(beta)-D-ribofuranosyl 5'-monophosphate synthetase (purP) (EC 6.3.4.-)	CL2	Yes	no change	COG1759R
MA0216	398	20089114	S-adenosylmethionine synthetase (mat)	Archaeal S-adenosylmethionine synthetase (EC 2.5.1.6) (Methionine adenosyltransferase) (AdoMet synthetase)	CL2	Yes	no change	COG1812E

MA0217	289	20089115	ATP phosphoribosyltransferase (hisG)	ATP phosphoribosyltransferase (hisG) (EC 2.4.2.17)	CL3	Yes	no change	COG0040E
MA0218	245	20089116	1-(5-phosphoribosyl)-5-	1-(5-phosphoribosyl)-5-((5-phosphoribosylamino)methylideneamino) imidazole-4-carboxamide isomerase (hisA) (EC 5.3.1.16)	CL2	Yes	no change	COG0106E
MA0219	191	20089117	imidazoleglycerol-phosphate dehydratase	Imidazoleglycerol-phosphate dehydratase (hisB) (EC 4.2.1.19)	CL2	Yes	no change	COG0131E
MA0241	448	20089139	phosphomannomutase	Phosphoglucomutase/Phosphomannomutase	CL2	Yes	more specific	COG1109G
MA0245	401	20089143	hypothetical protein MA0245	protein of unknown function DUF521	CL4	nd	more specific	COG1679S
MA0246	422	20089144	4-hydroxybenzoate decarboxylase	3-octaprenyl-4-hydroxybenzoate carboxy-lyase (ubiD) (EC 4.1.1.-)	CL3	Yes	more specific	COG0043H
MA0258	370	20089156	transposase	transposase	CL4	Yes	no change	COG0675L
MA0261	415	161484942	thiamine biosynthesis protein ThiC	Thiamine biosynthesis protein thiC (thiC)	CL2	nd	no change	COG0422H
MA0266	500	20089164	hypothetical protein MA0266	UPF0027-domain protein	CL4	nd	more specific	COG1690S
MA0269	316	20089167	tetrahydromethanopterin S-methyltransferase subunit H (mtrH)	Tetrahydromethanopterin S-methyltransferase subunit H (mtrH) (EC 2.1.1.86)	CL2	Yes	no change	COG1962H
MA0270	73	20089168	tetrahydromethanopterin S-methyltransferase subunit G (mtrG)	Tetrahydromethanopterin S-methyltransferase subunit G (EC 2.1.1.86)	CL2	Yes	no change	COG4064H
MA0271	74	20089169	tetrahydromethanopterin S-methyltransferase subunit F (mtrF)	Tetrahydromethanopterin S-methyltransferase subunit F (EC 2.1.1.86)	CL2	nd	no change	COG4218H
MA0272	240	20089170	tetrahydromethanopterin S-methyltransferase subunit A (mtrA)	Tetrahydromethanopterin S-methyltransferase subunit A (mtrA) (EC 2.1.1.86)	CL2	Yes	no change	COG4063H
MA0274	267	20089172	tetrahydromethanopterin S-methyltransferase subunit C (mtrC)	Tetrahydromethanopterin S-methyltransferase subunit C (mtrC) (EC 2.1.1.86)	CL2	Yes	no change	COG4061H
MA0275	249	20089173	tetrahydromethanopterin S-methyltransferase subunit D (mtrD)	Tetrahydromethanopterin S-methyltransferase subunit D (mtrD) (EC 2.1.1.86)	CL2	Yes	no change	COG4060H
MA0276	304	20089174	tetrahydromethanopterin S-methyltransferase subunit E (mtrE)	Tetrahydromethanopterin S-methyltransferase subunit E (mtrE) (EC 2.1.1.86)	CL2	Yes	no change	COG4059H
MA0279	639	20089177	sodium:proline symporter (proline permease) (purP)	sodium/solute symporter	CL3	Yes	less specific	COG0591ER

MA0306	584	20089204	formylmethanofuran dehydrogenase, subunit A	Molybdenum formylmethanofuran dehydrogenase subunit A (fmdA) (EC 1.2.99.5)	CL2	Yes	no change	COG1229C
MA0308	129	20089206	formylmethanofuran dehydrogenase, subunit D	Molybdenum formylmethanofuran dehydrogenase subunit D (fmdD) (EC 1.2.99.5)	CL2	Yes	no change	COG1153C
MA0309	433	20089207	formylmethanofuran dehydrogenase, subunit B	Molybdenum formylmethanofuran dehydrogenase subunit B (fmdB) (EC 1.2.99.5)	CL2	Yes	no change	COG1029C
MA0311	256	20089209	outer membrane protein	Protein of unknown function DUF541	CL4	nd	more specific	COG2968S
MA0345	131	20089243	hypothetical protein MA0345	hypothetical protein	CL5	nd	no change	COG4744S
MA0385	1478	161484941	cobaltochelatase (cobN)	magnesium/cobalt chelatase-domain containing protein	CL4	Yes	more specific	COG1429H
MA0431	60	20089323	ferredoxin	4Fe-4S ferredoxin, iron-sulfur protein	CL2	nd	more specific	COG1145C
MA0439	267	20089331	fructose-bisphosphate aldolase	2-amino-3,7-dideoxy-D-threo-hept-6-ulosonate synthase CL2	CL2	Yes	more specific	COG1830G
MA0440	171	20089332	deoxyuridine 5'-triphosphate nucleotidohydrolase	Deoxyuridine 5'-triphosphate nucleotidohydrolase (dUTPase) (EC 3.6.1.23)	CL2	Yes	no change	COG0717F
MA0455	461	20089346	methanol-5-hydroxybenzimidazolylcobamide co-methyltransferase, isozyme 1	methanol corrinoid protein (mtaB)	CL1	Yes	no change	-
MA0456	258	20089347	methanol-5-hydroxybenzimidazolylcobamide co-methyltransferase, isozyme 1	methanol corrinoid protein (mtaC)	CL1	Yes*	less specific	COG5012R
MA0488	923	20089377	cell surface protein	Hypothetical protein	CL5	nd	less specific	COG0823U, COG3291R
MA0490	848	20089379	sensory transduction histidine kinase	Multisensor signal transduction histidine kinase	CL4	Yes	more specific	COG2202T, COG3920T
MA0493	216	20089382	hypothetical protein MA0493	pyridoxal 5'-phosphate-dependent-protein, UPF0001family	CL4	nd	more specific	COG0325R
MA0507	172	20089396	antibiotic resistance protein McbG	Protein with pentapeptide repeats	CL4	nd	less specific	COG1357S
MA0509	482	20089398	surface antigen gene	NHL repeat domain (Beta propeller clan) protein	CL4	nd	more specific	COG3391S
MA0511	204	20089400	hypothetical protein MA0511	protein of unknown function with UPF0228 domain	CL4	nd	more specific	-
MA0523	900	20089412	DNA mismatch repair protein (mutS)		CL2	Yes	no change	COG0249L
MA0527	213	20089416	dimethylamine corrinoid protein	dimethylamine corrinoid protein (mtbC)	CL2	nd	no change	COG5012R

MA0528	495	20089417	trimethylamine methyltransferase	Trimethylamine methyltransferase (mttB) (EC 2.1.1.-)	CL2	Yes	no change	COG5598H
MA0529	216	20089418	trimethylamine corrinoid protein	trimethylamine corrinoid protein	CL2	Yes	no change	COG5012R
MA0530	353	20089419	transmembrane protein MttP	DUF6-domain membrane protein	CL4	nd	more specific	COG0697GER
MA0531	101	20089420	hypothetical protein MA0531	hypothetical protein	CL5	nd	no change	-
MA0534	533	20089423	lysyl-tRNA synthetase (lysK)	Lysyl-tRNA synthetase (EC 6.1.1.6)	CL2	Yes	no change	COG1384J
MA0540	95	20089429	hypothetical protein MA0540	Protein with MazG nucleotide pyrophosphohydrolase domain	CL4	Yes	more specific	COG1694R
MA0541	273	20089430	imidazole glycerol phosphate synthase subunit HisF	Imidazoleglycerol-phosphate synthase subunit hisF (cyclase subunit) (EC 4.1.3.-)	CL2	Yes	no change	COG0107E
MA0550	365	20089439	chorismate synthase (aroC)	Chorismate synthase (aroC) (EC 4.2.3.5)	CL2	Yes	no change	COG0082E
MA0551	1349	20089440	sensory transduction histidine kinase	Multisensor signal transduction histidine kinase	CL4	nd	more specific	COG2202T, COG3920T
MA0558	748	20089447	hypothetical protein MA0558	Hypothetical protein	CL5	nd	no change	-
MA0561	791	20089450	hypothetical protein MA0561	Hypothetical protein	CL5	Yes	no change	-
MA0563	399	20089452	hypothetical protein MA0563	Hypothetical protein	CL5	Yes	no change	COG2834M
MA0564	423	20089453	hypothetical protein MA0564	Hypothetical protein	CL5	nd	no change	-
MA0565	221	20089454	hypothetical protein MA0565	Hypothetical protein	CL5	nd	no change	-
MA0566	318	20089455	hypothetical protein MA0566	Hypothetical protein	CL5	nd	no change	-
MA0573	349	20089462	heme d1 biosynthesis protein NirJ	Radical SAM family protein	CL4	Yes	more specific	COG0535R
MA0575	158	20089464	heme d1 biosynthesis protein NirH	Transcriptional regulator	CL4	nd	less specific	COG1522K
MA0578	324	20089467	delta-aminolevulinic acid dehydratase	Delta-aminolevulinic acid dehydratase (Porphobilinogen synthase) (ALAD) (ALADH) (hemB) (EC 4.2.1.24)	CL2	Yes	no change	COG0113H
MA0581	424	20089470	glutamate-1-semialdehyde aminotransferase	Glutamate-1-semialdehyde 2,1-aminomutase (hemL) (EC 5.4.3.8)	CL2	Yes	no change	COG0001H
MA0583	311	20089472	dihydroorotate dehydrogenase 1B	Dihydroorotate dehydrogenase, catalytic subunit (Dihydroorotate oxidase) (pyrD) (EC 1.3.3.1)	CL2	Yes	more specific	COG0167F
MA0587	579	20089476	glutamyl-tRNA synthetase (gltX)	Glutamyl-tRNA synthetase (EC 6.1.1.17)	CL3	Yes	no change	COG0008J
MA0592	523	20089481	D-3-phosphoglycerate dehydrogenase	D-3-phosphoglycerate dehydrogenase (serA) (PGDH) (EC 1.1.1.95)	CL2	Yes	no change	COG0111HE
MA0595	138	20089484	50S ribosomal protein L18e	LSU ribosomal protein L18E (rpl18e)	CL2	Yes	no change	COG1727J
MA0596	140	161484940	50S ribosomal protein L13P	LSU ribosomal protein L13P (rpl13p)	CL2	Yes	no change	COG0102J

MA0597	134	20089486	30S ribosomal protein S9P	SSU ribosomal protein S9P (rps9p)	CL2	Yes	no change	COG0103J
MA0598	62	20089487	DNA-directed RNA polymerase subunit N (rpoN)	DNA-directed RNA polymerase subunit N	CL2	nd	no change	COG1644K
MA0600	247	20089489	30S ribosomal protein S2	SSU ribosomal protein S2P (rps2p)	CL2	Yes	no change	COG0052J
MA0604	365	20089493	isopentenyl pyrophosphate isomerase	Isopentenyl-diphosphate delta-isomerase (EC 5.3.3.2)	CL2	Yes	more specific	COG1304C
MA0605	447	20089494	metallo-beta-lactamase	UPF0036 protein, beta-lactamase-like	CL4	Yes*	less specific	COG0595R
MA0606	324	20089495	bifunctional short chain isoprenyl diphosphate synthase	Bifunctional short chain isoprenyl diphosphate synthase (Includes: Farnesyl pyrophosphate synthetase (EC 2.5.1.1) (FPP synthetase) (Dimethylallyltranstransferase); Geranyltranstransferase (EC 2.5.1.10)) (idsA)	CL2	Yes	no change	COG0142H
MA0608	883	20089497	pyruvate phosphate dikinase (ppdk)	Pyruvate phosphate dikinase EC 2.7.9.1	CL2	Yes	no change	COG0574G
MA0610	337	20089499	transcription initiation factor IIB	Transcription initiation factor IIB (TFIIB)	CL2	nd	no change	COG1405K
MA0619	654	20089507	sensory transduction histidine kinase	Multisensor signal transduction histidine kinase	CL4	Yes	more specific	COG2202T, COG3920T
MA0639	163	20089526	rubrerythrin	Rubrerythrin	CL2	Yes	no change	COG1592C
MA0641	467	20089528	hypothetical protein MA0641	protein with PHP (polymerase and histidinol phosphatase) C-terminal domain	CL4	nd	more specific	COG1379S
MA0644	271	20089531	translation initiation factor IF-2 subunit alpha	translation initiation factor IF-2 subunit alpha	CL2	Yes	no change	COG1093J
MA0645	62	20089532	30S ribosomal protein S27e	SSU ribosomal protein S27E (rps27e)	CL2	Yes	no change	COG2051J
MA0646	92	20089533	50S ribosomal protein L44e	LSU ribosomal protein L44E (rpl44e)	CL2	Yes	no change	COG1631J
MA0648	414	20089535	DNA primase small subunit	DNA primase, small subunit	CL3	Yes	no change	COG1467L
MA0651	788	20089538	hypothetical protein MA0651	FbpA, DUF814 containing protein	CL4	Yes	more specific	COG1293K
MA0663	199	20089550	Na+-transporting NADH:ubiquinone oxidoreductase, subunit 5 (nqr5)	Electron transport complex protein rnfA (Nitrogen fixation protein rnfA)	CL2	Yes	more specific	COG4657C

MA0664	264	20089551	ferredoxin (nqr6)	electron transport complex rnfB-like protein (Nitrogen fixation protein rnfB)	CL2	Yes	more specific	COG1145C, COG2878C
MA0672	69	20089557	histone	archaeal histone A (hmaA)	CL2	nd	more specific	COG2036B
MA0674	572	20089559	pyruvate carboxylase subunit B	pyruvate carboxylase subunit B (EC 6.4.1.1) (pycB)	CL2	Yes	no change	COG0511I, COG1038C, COG5016C
MA0675	493	20089560	pyruvate carboxylase subunit A	pyruvate carboxylase subunit A (EC 6.4.1.1) (pycA)	CL2	Yes	no change	COG0439I
MA0681	701	20089566	Mcm2 DNA replication licensing factor	MCM (minichromosome maintenance protein)	CL2	Yes	no change	COG1241L
MA0684	127	20089569	hypothetical protein MA0684 (fprB)	Rubrerythrin-related protein	CL4	Yes	more specific	COG1592C
MA0685	228	20089570	sulfite reductase	Nitrite/sulphite reductase-related protein	CL4	Yes	less specific	COG1251C
MA0688	409	20089573	heterodisulfide reductase, subunit D	CoB-CoM heterodisulfide reductase, subunit D (EC 1.8.98.1) (hdrD)	CL2	Yes	no change	COG0247C
MA0689	211	20089574	hypothetical protein MA0689	protein of unknown function DUF116	CL4	Yes	more specific	COG1852S
MA0691	389	20089576	geranylgeranyl reductase	thioredoxin reductase (NADPH)	CL2	nd	more specific	COG0644C
MA0711	183	20089596	phenylacrylic acid decarboxylase		CL2	Yes	no change	COG0163H
MA0719	197	20089604	hypothetical protein MA0719	RNA methyltransferase (EC 2.1.1.-)	CL2	Yes	more specific	COG2263J
MA0722	386	20089607	Sep-tRNA:Cys-tRNA synthetase	Sep-tRNA:Cys-tRNA synthase (EC 2.5.1.-)	CL3	Yes	no change	COG1103R
MA0726	420	20089611	diaminopimelate decarboxylase (lysA)	Orn/DAP/Arg decarboxylase family / TabA-like	CL3	Yes	less specific	COG0019E
MA0759	1281	20089644	sensory transduction histidine kinase	Hypothetical protein	CL5	nd	less specific	COG2202T, COG3920T
MA0762	955	20089647	ATP-dependent DNA helicase	DEAD/DEAH box helicase-like protein	CL3	nd	less specific	COG1201R
MA0788	156	20089672	glutamine ABC transporter, ATP-binding protein (glnQ)	Phosphate import ABC transporter ATPase subunit (EC 3.6.3.27)	CL2	nd	more specific	COG1126E
MA0807	220	20089691	NifU family protein	Nitrogen-fixing NifU-like protein	CL4	nd	less specific	COG0822C
MA0808	384	20089692	homocysteine desulfhydrase	Cysteine desulfurase	CL2	Yes	more specific	COG1104E
MA0809	139	20089693	hypothetical protein MA0809	Hypothetical protein	CL5	Yes	no change	COG2210S
MA0810	75	20089694	hypothetical protein MA0810	response regulator SirA-like protein, UPF0033 family	CL4	nd	more specific	COG0425O
MA0815	317	20089699	tyrosyl-tRNA synthetase (tyrS)	Tyrosyl-tRNA synthetase (EC 6.1.1.1)	CL2	Yes	no change	COG0162J

MA0818	85	20089702	hypothetical protein MA0818	Protein with PRC-barrel domain	CL4	Yes	more specific	COG1873S
MA0823	476	20089707	ss-DNA-specific exonuclease	Single stranded DNA specific Exonuclease recJ	CL3	nd	no change	COG0608L
MA0825	172	20089709	hypothetical protein MA0825	Protein of unknown function DUF1699	CL4	nd	more specific	-
MA0826	547	20089710	histone acetyltransferase	Elongator complex protein 3, elp3	CL2	Yes	more specific	COG1243KB
MA0829	671		hypothetical protein MA0829	S-layer protein	CL1	Yes	more specific	
MA0849	563	20089733	hypothetical protein MA0849	putative methylamine methyltransferase corrinoid activation protein	CL4	nd	more specific	COG1145C, COG3894R
MA0850	2275	20089734	cell surface protein	magnesium/cobalt chelatase-domain containing protein	CL4	nd	more specific	COG1520S, COG3291R, COG3420P
MA0863	749	20089747	sensory transduction histidine kinase	pleC (Non-motile and phage-resistance protein) -like multisensor signal transduction histidine kinase	CL4	nd	no change	COG0642T, COG2202T, COG2203T
MA0865	67	20089749	hypothetical protein MA0865	Hypothetical protein	CL5	nd	no change	-
MA0887	317	20089771	phosphate ABC transporter, solute-binding protein	Phosphate import ABC transporter, phosphate binding protein (pstS)	CL3	Yes	no change	COG0226P
MA0888	296	20089772	phosphate ABC transporter, permease protein	Phosphate import ABC transporter permease protein (pstC)	CL3	Yes	no change	COG0573P
MA0889	307	20089773	phosphate ABC transporter, permease protein	Phosphate import ABC transporter permease protein (pstA)	CL3	Yes	no change	COG0581P
MA0890	258	20089774	phosphate ABC transporter, ATP-binding protein (pstB)	CBS (Cystathionine-beta-synthase) and DUF293 domains-containing protein	CL3	Yes	less specific?	COG1117P
MA0895	111	20089779	translation initiation factor IF-1A	translation initiation factor aIF-1A	CL2	Yes	no change	COG0361J
MA0902	113	20089781	nascent polypeptide-associated complex protein	Nascent polypeptide-associated complex (NAC), chaperone protein	CL2	Yes	no change	COG1308K
MA0908	120	20089786	phosphoribosyl-AMP cyclohydrolase	Phosphoribosyl-AMP cyclohydrolase (hisI) (EC 3.5.4.19)	CL2	nd	no change	COG0139E
MA0913	202	20089791	imidazole glycerol phosphate synthase subunit HisH	(EC 2.4.2.-)	CL2	Yes	no change	COG0118E
MA0914	454	20089792	hypothetical protein MA0914	AIR synthase related protein	CL4	Yes	more specific	COG1973O
MA0916	265	20089794	erythrocyte band 7 integral membrane protein	SPFH domain / Band 7 family integral membrane protein	CL4	Yes	less specific	COG0330O
MA0923	125	20089801	30S ribosomal protein S8e	SSU ribosomal protein S8E (rps8e)	CL2	Yes	no change	COG2007J

MA0924	163	20089802	leucine responsive regulatory protein	HTH transcriptional regulator, AsnC/Lrp family	CL3	Yes	less specific	COG1522K
MA0927	227	20089805	hypothetical protein MA0927		CL5	nd	no change	COG1549J
MA0928	98	20089806	hypothetical protein MA0928	hypothetical protein	CL5	nd	no change	-
MA0929	281	20089807	transmembrane protein MttP	DUF6-domain membrane protein	CL4	nd	more specific	COG0697GER
MA0930	70	20089808	transmembrane protein MttP	DUF6-domain membrane protein	CL4	nd	more specific	-
MA0931	217	20089809	trimethylamine corrinoid protein	trimethylamine corrinoid protein	CL2	nd	no change	COG5012R
MA0932	495	20089810	trimethylamine methyltransferase	Trimethylamine methyltransferase (mttB) (EC 2.1.1.-)	CL2	nd	no change	COG5598H
MA0934	218	20089812	dimethylamine corrinoid protein	dimethylamine corrinoid protein (mtbC)	CL2	nd	no change	COG5012R
MA0937	235	20089815	ABC transporter, ATP-binding protein	atypical ABC-ATPase SufC	CL3	Yes	no change	COG0396O
MA0940	183	20089818	hypothetical protein MA0940	alpha-ribazole-phosphate phosphatase (cobZ) (EC 3.1.3.73)	CL2	Yes	more specific	COG1267I
MA0949	152	20089827	30S ribosomal protein S15P	SSU ribosomal protein S15P (rps15p)	CL2	Yes	no change	COG0184J
MA0951	356	20089829	hemin permease (hemU)	Iron complex/Vitamin B12 ABC transporter permease protein	CL3	Yes	more specific	COG0609P
MA0953	407	20089831	cell division protein FtsZ	Tubulin/FtsZ family protein	CL4	nd	less specific	COG0206D
MA0954	422	20089832	hypothetical protein MA0954	protein of unknown function DUF11	CL4	nd	more specific	COG1361M, COG2373R
MA0963	247	20089841	hypothetical protein MA0963	Protein of unknown function UPF0278	CL4	Yes	more specific	COG1458R
MA0969	243	20089846	orotidine-5-phosphate decarboxylase	Orotidine 5'-phosphate decarboxylase (OMP decarboxylase) (PyrF) (EC 4.1.1.23)	CL2	nd	no change	COG0284F
MA0984	383	20089861	hypothetical protein MA0984	hypothetical protein	CL5	nd	no change	COG3385L
MA1009	370	20089885	transposase	transposase	CL4	Yes	no change	COG0675L
MA1011	468	20089886	acetyl-CoA decarbonylase/synthase complex subunit gamma	Acetyl-CoA decarbonylase/synthase complex gamma subunit (EC 2.1.1.-)	CL2	Yes	no change	COG1456C
MA1012	436	20089887	acetyl-CoA decarbonylase/synthase complex subunit delta	Acetyl-CoA decarbonylase/synthase complex delta subunit	CL2	Yes	no change	COG2069C
MA1013	232	20089888	carbon-monoxide dehydrogenase accessory protein	carbon monoxide dehydrogenase/acetyl-CoA synthase complex, nickel-inserting subunit	CL3	Yes	no change	COG3640D

MA1014	469	20089889	acetyl-CoA decarbonylase/synthase complex subunit beta	Acetyl-CoA decarbonylase/synthase complex beta subunit (EC 2.3.1.-)	CL2	Yes	no change	COG1614C
MA1016	806	20089891	acetyl-CoA decarbonylase/synthase complex subunit alpha	Acetyl-CoA decarbonylase/synthase complex alpha subunit 2 (EC 1.2.99.2)	CL2	Yes	no change	COG1152C
MA1018	335	20089893	glyceraldehyde-3-phosphate dehydrogenase	glyceraldehyde-3-phosphate dehydrogenase	CL2	Yes	no change	COG0057G
MA1022	605	20089897	indolepyruvate ferredoxin oxidoreductase, subunit alpha	Protein containing N-terminal Pyruvate flavodoxin/ferredoxin oxidoreductase region, C-terminal TPP-binding region and ferredoxin domain	CL4	nd	less specific	COG4231C
MA1041	73	20089913	hypothetical protein MA1041	cobalt ABC transporter ATP-binding protein cbiO	CL3	nd	more specific	COG0675L
MA1072	337	20089942	50S ribosomal protein L3P	LSU ribosomal protein L3P (rpl3p)	CL2	Yes	no change	COG0087J
MA1073	253	20089943	50S ribosomal protein L4P	LSU ribosomal protein L4P (rpl4p)	CL2	Yes	no change	COG0088J
MA1074	82	20089944	50S ribosomal protein L23	LSU ribosomal protein L23P (rpl23p)	CL2	Yes	no change	COG0089J
MA1075	238	20089945	50S ribosomal protein L2P	LSU ribosomal protein L2P (rpl2p)	CL2	Yes	no change	COG0090J
MA1076	136	20089946	30S ribosomal protein S19P	SSU ribosomal protein S19P (rps19p)	CL2	Yes	no change	COG0185J
MA1077	151	20089947	50S ribosomal protein L22P	LSU ribosomal protein L22P (rpl22p)	CL2	Yes	no change	COG0091J
MA1079	67	20089949	50S ribosomal protein L29	LSU ribosomal protein L29P (rpl29P)	CL2	Yes	no change	COG0255J
MA1080	110	20089950	ribonuclease P protein component 1	Ribonuclease P protein component 1 (rnp1) (EC 3.1.26.5)	CL2	Yes	no change	COG1588J
MA1081	109	20089951	30S ribosomal protein S17P	SSU ribosomal protein S17P (rps17p)	CL2	Yes	no change	COG0186J
MA1082	132	20089952	50S ribosomal protein L14P	LSU ribosomal protein L14P (rpl14p)	CL2	Yes	no change	COG0093J
MA1085	165	20089955	50S ribosomal protein L5P	LSU ribosomal protein L5P (rpl5p)	CL2	Yes	no change	COG0094J
MA1086	50	20089956	30S ribosomal protein S14P	SSU ribosomal protein S14P (rps14p)	CL2	nd	no change	COG0199J
MA1087	130	20089957	30S ribosomal protein S8P	SSU ribosomal protein S8P (rps8p)	CL2	Yes	no change	COG0096J
MA1089	161	20089959	50S ribosomal protein L32e	LSU ribosomal protein L32E (rpl32e)	CL2	Yes	no change	COG1717J
MA1090	151	20089960	50S ribosomal protein L19e	LSU ribosomal protein L19E (rpl19e)	CL2	Yes	no change	COG2147J

MA1091	174	20089961	50S ribosomal protein L18P	LSU ribosomal protein L18P (rpl18p)	CL2	Yes	no change	COG0256J
MA1092	209	20089962	30S ribosomal protein S5P	SSU ribosomal protein S5P (rps5p)	CL2	Yes	no change	COG0098J
MA1093	153	20089963	50S ribosomal protein L30P	LSU ribosomal protein L30P (rpl30p)	CL2	Yes	no change	COG1841J
MA1095	491	20089965	preprotein translocase subunit SecY	Preprotein translocase SecY subunit	CL2	Yes	no change	COG0201U
MA1103	206	20089972	integral membrane protein	Integral membrane protein DUF106	CL4	Yes	more specific	COG1422S
MA1108	182	20089977	30S ribosomal protein S13P	SSU ribosomal protein S13P (rps13p)	CL2	Yes	no change	COG0099J
MA1110	126	20089979	30S ribosomal protein S11P	SSU ribosomal protein S11P (rps11p)	CL2	Yes	no change	COG0100J
MA1118	406	20089984	ABC transporter, permease protein	protein of unknown function DUF214	CL4	nd	less specific	COG0577V
MA1120	312	20089986	ABC transporter, ATP-binding protein	ABC transporter, ATPase subunit	CL2	nd	no change	COG1136V
MA1152	362	20090018	fructose 1,6-bisphosphatase II	Fructose 1-6-bisphosphatase	CL2	Yes	no change	COG1494G
MA1167	283	20090033	ribose-phosphate pyrophosphokinase	Ribose-phosphate pyrophosphokinase (RPPK) (Phosphoribosyl pyrophosphate synthetase) (PRPP synthetase) (EC 2.7.6.1)	CL2	Yes	no change	COG0462FE
MA1168	158	20090034	putative molybdenum cofactor biosynthesis protein C	Molybdenum cofactor biosynthesis protein C (moaC)	CL2	nd	more specific	COG0315H
MA1185	311	20090051	UDP-glucose 4-epimerase	UDP-galactose -4-epimerase	CL3	Yes	more specific	COG0451MG
MA1193	316	20090059	hypothetical protein MA1193	hypothetical protein	CL5	nd	no change	COG1665S
MA1223	164	20090087	3-isopropylmalate dehydratase	3-Isopropylmalate dehydratase small subunit (3-isopropylmalate isomerase small subunit) and homoaconitase small subunit - dual specificity	CL2	nd	more specific	COG0066E
MA1237	276	20090101	molybdate ABC transporter, solute-binding protein	molybdenum ABC transporter, molybdate-binding protein (modA)	CL3	nd	no change	COG0725P
MA1241	441	20090105	formylmethanofuran dehydrogenase, subunit B	Molybdenum formylmethanofuran dehydrogenase subunit B (fmdB) (EC 1.2.99.5)	CL2	nd	no change	COG1029C
MA1255	102	20090119	30S ribosomal protein S10P	SSU ribosomal protein S10P (rps10p)	CL2	Yes	no change	COG0051J

MA1256	422	20090120	elongation factor 1-alpha	translation elongation factor EF-1, subunit alpha (EF-1A, EF-Tu)	CL2	Yes	no change	COG5256J
MA1257	730	20090121	elongation factor EF-2	translation elongation factor EF-2	CL1	Yes	no change	COG0480J
MA1258	189	20090122	30S ribosomal protein S7P	SSU ribosomal protein S7P (rps7p)	CL2	Yes	no change	COG0049J
MA1259	142	20090123	30S ribosomal protein S12P	SSU ribosomal protein S12P (rps12p)	CL2	Yes	no change	COG0048J
MA1261	99	20090125	50S ribosomal protein L30e	LSU ribosomal protein L30E (rpl30e)	CL2	Yes	no change	COG1911J
MA1262	397	20090126	DNA-directed RNA polymerase subunit A''	DNA-directed RNA polymerase subunit A'', rpoA2	CL2	Yes	no change	COG0086K
MA1263	880	20090127	DNA-directed RNA polymerase subunit alpha	DNA-directed RNA polymerase subunit A', rpoA1	CL2	Yes	no change	COG0086K
MA1264	604	20090128	DNA-directed RNA polymerase subunit B'	DNA-directed RNA polymerase subunit B', rpoB1	CL2	nd	no change	COG0085K
MA1265	550	20090129	DNA-directed RNA polymerase subunit beta''	DNA-directed RNA polymerase subunit B'', rpoB2	CL2	Yes	no change	COG0085K
MA1266	78	20090130	DNA-directed RNA polymerase subunit H	DNA-directed RNA polymerase subunit H, rpoH	CL2	Yes	no change	COG2012K
MA1267	616	20090131	sensory transduction histidine kinase	Multisensor signal transduction histidine kinase	CL4	nd	more specific	COG0784T, COG2202T, COG3920T
MA1275	411	20090139	S-adenosyl-L-homocysteine hydrolase	Adenosylhomocysteinase (ahcY) (EC 3.3.1.1)	CL2	Yes	no change	COG0499H
MA1276	442	20090140	N-ethylammeline chlorohydrolase	metal-dependent hydrolase	CL3	Yes	less specific	COG0402FR
MA1284	339	20090148	hypothetical protein MA1284	Hypothetical protein	CL5	nd	no change	-
MA1288	272	20090152	23S RNA methyltransferase J	ribosomal RNA large subunit methyltransferase RrmJ/FtsJ (EC 2.1.1.-)	CL2	Yes	no change	COG0293J, COG3269R
MA1289	218	20090153	hypothetical protein MA1289	Hypothetical protein with PHP domain	CL4	nd	more specific	COG1387ER
MA1291	218	20090155	hypothetical protein MA1291	Hypothetical protein with PHP domain	CL4	nd	more specific	COG1387ER
MA1318	491	20090180	argininosuccinate lyase	Argininosuccinate lyase (EC 4.3.2.1) (Arginosuccinase)	CL2	Yes	no change	COG0165E
MA1322	1428	20090183	sensory transduction histidine kinase	pleD (response regulator)-like multisensor signal transduction histidine kinase	CL4	nd	no change	COG2202T, COG3920T
MA1335	76	20090196	hypothetical protein MA1335	N-acetylglucosamine-1-phosphate uridyltransferase (EC 2.7.7.23)	CL3	nd	more specific	-

MA1344	81	20090205	hypothetical protein MA1344	hypothetical protein	CL5	nd	no change	COG4895S
MA1351	105	20090212	translation initiation factor IF-1A	translation initiation factor aIF-1A	CL2	nd	no change	COG0361J
MA1353	110	20090214	translation initiation factor IF-1A	translation initiation factor aIF-1A	CL2	nd	no change	COG0361J
MA1363	284	20090224	phosphoglycolate phosphatase	HAD-superfamily hydrolase	CL4	nd	less specific	COG1011R
MA1370	266	20090231	diphthine synthase	diphthine synthase (EC 2.1.1.98)	CL2	Yes	no change	COG1798J
MA1372	129	20090233	hypothetical protein MA1372	DUF555-containing protein	CL4	Yes	more specific	COG1885S
MA1376	131	20090237	phosphoribosylaminoimidazole carboxylase	Phosphoribosylaminoimidazole carboxylase catalytic subunit (AIR carboxylase) (purE) (EC 4.1.1.21)	CL2	Yes	no change	COG0041F
MA1381	213	20090242	GTP-binding protein	GTP-binding domain protein	CL4	Yes	less specific	COG1100R
MA1382	119	20090243	hypothetical protein MA1382	protein of unknown function UCP004977	CL4	Yes	more specific	COG3365S
MA1384	216	20090245	6-phospho-3-hexuloisomerase	6-phospho-3-hexuloisomerase	CL2	Yes	no change	COG0794M
MA1385	370	20090246	aspartate aminotransferase	Aspartate aminotransferase (EC 2.6.1.1)	CL2	Yes	no change	COG0436E
MA1391	291	20090252	hypothetical protein MA1391	CBS (Cystathionine-beta-synthase) and DUF293 domains-containing protein	CL4	Yes	more specific	COG2524K
MA1393	420	20090254	3-isopropylmalate dehydratase	Homoaconitase large subunit (EC 4.2.1.36) (homoaconitate hydratase) (hacA)	CL2	Yes	more specific	COG0065E
MA1396	167	20090257	hypothetical protein MA1396	amino acid binding-domain protein	CL4	nd	more specific	COG2150R
MA1422	550	20090282	acyl-CoA synthetase	fatty-acid--CoA ligase (Acyl-CoA synthetase) (EC 6.2.1.-)	CL3	nd	no change	COG0318IQ
MA1423	151	20090283	hypothetical protein MA1423	thioesterase superfamily protein	CL4	Yes	more specific	COG0824R
MA1424	192	20090284	transcriptional regulator	HTH-type transcriptional regulator	CL3	Yes	more specific	COG1396K
MA1425	477	20090285	transposase	transposase	CL4	Yes	no change	COG3436L
MA1435	399	20090295	potassium channel protein	protein with PhoU and TrkA-C domains	CL4	Yes	more specific	COG3273S
MA1442	197	20090301	hypothetical protein MA1442	DUF655 containing protein	CL4	Yes	more specific	COG1491J
MA1446	97	20090305	50S ribosomal protein L21e	LSU ribosomal protein L21E (rpl21e)	CL2	Yes	no change	COG2139J
MA1447	431	20090306	hypothetical protein MA1447	THUMP-domain containing protein	CL4	nd	more specific	COG1258J
MA1460	246	20090319	phage shock protein A	Phage shock protein A PspA/IM30	CL4	Yes	no change	COG1842KT
MA1468	147	20090327	response regulator receiver	Response regulator receiver	CL4	nd	no change	COG0784T
MA1469	137	20090328	response regulator receiver	Response regulator receiver	CL4	Yes	no change	COG0784T
MA1471	89	20090330	elongation factor 1-beta	translation elongation factor EF-1 beta (EF-1-beta)	CL2	Yes	no change	COG2092J
MA1478	617	20090337	heat shock protein 70	Chaperone DnaK (Hsp70)	CL2	Yes	no change	COG0443O
MA1482	448	20090341	potassium transporter peripheral membrane component	Potassium uptake protein TrkA	CL2	Yes	no change	COG0569P

MA1484	407	20090343	geranylgeranyl reductase	Oxidoreductase with FAD binding domain	CL4	Yes	less specific	COG0644C
MA1485	102	20090344	ferredoxin	4Fe-4S ferredoxin, iron-sulfur protein	CL4	Yes	no change	COG1145C
MA1489	384	161484937	FO synthase subunit 2	Hypothetical protein	CL5	Yes*	less specific	COG1060HR
MA1495	124	20090354	F(420)H(2) dehydrogenase, subunit FpoA	F420H2 dehydrogenase subunit A	CL2	Yes	no change	COG0838C
MA1496	184	20090355	F(420)H(2) dehydrogenase, subunit FpoB	F420H2 dehydrogenase subunit B (fpoB)	CL2	Yes	no change	COG0377C
MA1497	158	20090356	NADH dehydrogenase subunit C	F420H2 dehydrogenase subunit C (fpoC)	CL2	Yes	more specific	COG0852C
MA1498	374	20090357	F(420)H(2) dehydrogenase, subunit FpoD	F420H2 dehydrogenase subunit D (fpoD)	CL2	Yes	no change	COG0649C
MA1499	348	20090358	F(420)H(2) dehydrogenase, subunit FpoH	F420H2 dehydrogenase subunit H	CL2	Yes	no change	COG1005C
MA1503	102	20090362	F(420)H(2) dehydrogenase, subunit FpoK	F420H2 dehydrogenase subunit K	CL2	Yes	no change	COG0713C
MA1504	672	20090363	F(420)H(2) dehydrogenase, subunit FpoL	F420H2 dehydrogenase subunit L	CL2	Yes	no change	COG1009CP
MA1505	495	20090364	F(420)H(2) dehydrogenase, subunit FpoM	F420H2 dehydrogenase subunit M	CL2	Yes	no change	COG1008C
MA1506	489	20090365	F(420)H(2) dehydrogenase, subunit FpoN	F420H2 dehydrogenase subunit N	CL3	Yes	no change	COG1007C
MA1521	120	20090380	50S ribosomal protein L7Ae	LSU ribosomal protein L7Ae (rpl7ae)	CL2	Yes	no change	COG1358J
MA1522	76	20090381	30S ribosomal protein S28e	SSU ribosomal protein S28E (rps28e)	CL2	Yes	no change	COG2053J
MA1523	62	20090382	50S ribosomal protein L24e	LSU ribosomal protein L24E (rpl24e)	CL2	Yes	no change	COG2075J
MA1524	151	20090383	nucleoside-diphosphate kinase (ndk)	Nucleoside diphosphate kinase (EC 2.7.4.6)	CL2	Yes	no change	COG0105F
MA1525	597	20090384	translation initiation factor IF-2 (infB)	translation initiation factor IF-2 (eIF-5B)	CL2	Yes	no change	COG0532J
MA1536	356	20090395	ABC transporter, ATP-binding protein	ABC transporter, ATPase subunit	CL2	nd	more specific	COG1131V
MA1546	294	20090404	hypothetical protein MA1546	Hypothetical protein	CL5	nd	no change	COG1277R
MA1547	301	20090405	hypothetical protein MA1547	Hypothetical protein	CL5	nd	no change	COG1277R
MA1548	524	20090406	hypothetical protein MA1548	protein with TolB-like six-bladed beta-propeller domain	CL5	nd	more specific	COG0823U
MA1549	159	20090407	hypothetical protein MA1549	NHL repeat domain (Beta propeller clan) protein	CL4	Yes	more specific	-
MA1550	140	20090408	hypothetical protein MA1550	protein with ion channel domain	CL4	nd	more specific	COG1226P

MA1554	138	20090412	hypothetical protein MA1554	pyridoxamine 5'-phosphate oxidase-related, FMN-binding	CL4	nd	more specific	COG3576R
MA1567	350	20090425	pyridoxine biosynthesis protein	Pyridoxal 5'-phosphate (Vitamin B6) synthase, lyase subunit pdxS	CL2	Yes	more specific	COG0214H
MA1583	929	20090441	DNA topoisomerase (ATP-hydrolyzing), subunit A	DNA gyrase subunit A	CL2	Yes	no change	COG0188L
MA1584	634	20090442	DNA topoisomerase (ATP-hydrolyzing), subunit B	DNA gyrase subunit B	CL2	nd	no change	COG0187L
MA1586	393	20090444	DNA topoisomerase VI subunit A	DNA topoisomerase, type VI subunit A	CL2	Yes	no change	COG1697L
MA1587	621	20090445	DNA topoisomerase VI subunit B	DNA topoisomerase, type VI subunit B	CL2	nd	no change	COG1389L
MA1589	203	20090447	hypothetical protein MA1589	protein of unknown function with UPF0228 domain	CL4	nd	more specific	-
MA1590	453	20090448	surface antigen gene	NHL repeat domain (Beta propeller clan) containing protein	CL4	nd	more specific	COG3391S
MA1591	443	20090449	surface antigen gene	NHL repeat domain (Beta propeller clan) protein	CL4	nd	more specific	COG3391S
MA1604	71	20090462	hypothetical protein MA1604	Hypothetical protein	CL5	nd	no change	-
MA1611	961	20090469	leucyl-tRNA synthetase	Leucyl-tRNA synthetase (EC 6.1.1.4)	CL2	Yes	no change	COG0495J
MA1614	195	20090472	hypothetical protein MA1614	protein with ADP-ribose binding-domain, UPF0189 family	CL4	nd	more specific	COG2110R
MA1615	339	20090473	methylcobalamin:coenzyme M methyltransferase (cmtM)	methanol-specific methylcobalamin:CoM methyltransferase (mtaA)	CL2	Yes	more specific	COG0407H
MA1616	461	20090474	methanol-5-hydroxybenzimidazolylcobamide co-methyltransferase, isozyme 3	methanol-5-hydroxybenzimidazolylcobamide co-methyltransferase (mtaB3) (EC 2.1.1.90)	CL1	Yes	no change	-
MA1617	260	20090475	methanol-5-hydroxybenzimidazolylcobamide co-methyltransferase, isozyme 3 (mtaC3)	corrinoid-containing methyl-accepting protein (mtaC3)	CL1	Yes*	more specific	COG5012R
MA1627	962	20090482	sensory transduction histidine kinase	Multisensor signal transduction histidine kinase	CL4	nd	no change	COG2202T, COG3920T
MA1628	899	20090483	sensory transduction histidine kinase	Multisensor signal transduction histidine kinase	CL4	nd	no change	COG2202T, COG3920T
MA1645	886	20090498	sensory transduction histidine kinase	GAF-containing Multisensor signal transduction histidine kinase	CL4	nd	more specific	COG2202T, COG3920T
MA1672	429	20090525	phosphopyruvate hydratase	Enolase (2-phosphoglycerate dehydratase) (EC 4.2.1.11)	CL2	Yes	no change	COG0148G

MA1678	839	20090530	H(+)-transporting ATPase	Plasma-membrane proton-efflux P-type ATPase (EC 3.6.3.6)	CL2	Yes	more specific	COG0474P
MA1684	444	20090536	aspartyl-tRNA synthetase	Aspartyl-tRNA synthetase (EC 6.1.1.12)	CL2	Yes	no change	COG0017J
MA1691	464	20090543	hypothetical protein MA1691	Protein of unknown function DUF711	CL4	nd	more specific	COG2848S
MA1696	111	20090548	hypothetical protein MA1696	Hypothetical protein	CL5	nd	no change	COG1196D
MA1708	231	20090560	uncharacterized Fe-S protein	Uncharacterized Fe-S protein	CL4	nd	no change	COG1600C
MA1710	321	20090562	N(5),N(10)-methenyltetrahydromethanopterin cyclohydrolase	methenyltetrahydromethanopterin cyclohydrolase (mch) (EC 3.5.4.27)	CL2	Yes	no change	COG3252H
MA1712	389	20090564	aspartate aminotransferase	Aminotransferase class I/II (EC 2.6.1.-)	CL3	Yes	less specific	COG0436E
MA1724	379	20090576	hypothetical protein MA1724	Small-conductance mechanosensitive ion channel	CL3	Yes	more specific	COG0668M
MA1725	435	20090577	phenylacetate-CoA ligase	Phenylacetate-coenzyme A ligase (EC 6.2.1.30)	CL2	Yes	no change	COG1541H
MA1726	203	20090578	indolepyruvate oxidoreductase subunit B	Indolepyruvate ferredoxin oxidoreductase subunit beta (iorB)	CL3	Yes	no change	COG1014C
MA1727	619	20090579	indolepyruvate ferredoxin oxidoreductase, subunit alpha	Protein containing N-terminal Pyruvate flavodoxin/ferredoxin oxidoreductase region, C-terminal TPP-binding region and ferredoxin domain	CL4	Yes	less specific	COG4231C
MA1736	160	20090588	Mov34 family protein	metal-dependent protease, JAMM-like	CL2	Yes	more specific	COG1310R
MA1752	224	20090604	ABC transporter, ATP-binding protein	Iron complex/Vitamin B12 ABC transporter ATPase subunit	CL3	nd	more specific	COG1136V
MA1759	466	20090610	hypothetical protein MA1759	hypothetical protein	CL5	nd	no change	-
MA1762	3988	20090613	cell surface protein	Protein containing N-terminal subtilase (Peptidase_S8) family domain + central repeat Fibronectin type III (fn3) domains + C-terminal repeat PDK domains	CL4	nd	more specific	COG1520S, COG3291R
MA1775	94	20090626	50S ribosomal protein L37Ae	LSU ribosomal protein L37Ae (rpl37ae)	CL2	Yes	no change	COG1997J
MA1778	230	20090629	putative RNA-associated protein	Ancient conserved region within archaeal exosome superoperon, UPF0023 domain protein	CL4	Yes	more specific	COG1500J
MA1779	247	20090630	proteasome subunit alpha	Proteasome alpha subunit (psmA) (EC 3.4.25.1)	CL2	Yes	no change	COG0638O

MA1784	196	20090635	50S ribosomal protein L15e	LSU ribosomal protein L15E (rpl15e)	CL2	Yes	no change	COG1632J
MA1786	301	20090637	transposase	transposase	CL4	nd	no change	COG0675L
MA1790	428	20090641	thiamine biosynthesis protein ThiC	Thiamine biosynthesis protein thiC (thiC)	CL2	Yes	no change	COG0422H
MA1802	553	20090653	dihydroxy-acid dehydratase (ilvD)	Dihydroxyacid dehydratase, EC 4.2.1.9	CL2	Yes	no change	COG0129EG
MA1806	280	20090657	methyltransferase	methyltransferase MtxX	CL3	Yes	no change	COG4002R
MA1817	154	20090668	riboflavin synthase (ribC)	riboflavin synthase	CL2	Yes	no change	COG1731H
MA1818	134	20090669	riboflavin synthase subunit beta	6,7-dimethyl-8-ribityllumazine synthase (ribH) (EC 2.5.1.9)	CL3	Yes	no change	COG0054H
MA1819	380	20090670	aspartate aminotransferase (aspB)	Aspartate aminotransferase (EC 2.6.1.1)	CL2	Yes	no change	COG0436E
MA1820	151	20090671	glycerol-3-phosphate cytidyltransferase (tagD)	Cytidyltransferase-related protein	CL4	Yes	less specific	COG0615MI
MA1821	500	20090672	hypothetical protein MA1821	CBS-domain and DUF39-domain containing protein	CL4	Yes	more specific	COG0517R, COG1900S
MA1838	919	20090688	cell surface protein	NHL repeat domain (Beta propeller clan) protein	CL4	nd	more specific	COG3291R, COG3391S
MA1904	1698	20090753	hypothetical protein MA1904	NHL repeat domain (Beta propeller clan) protein	CL4	nd	more specific	COG3291R, COG3391S
MA1956	545	20090804	phenylalanyl-tRNA synthetase subunit beta	Phenylalanyl-tRNA synthetase, beta subunit (EC 6.1.1.20)	CL2	nd	no change	COG0072J
MA1963	232	20090811	phosphoribosylformylglycinamidine synthase subunit I	phosphoribosylformylglycinamidine synthase I (FGAM synthase I) (purQ) (EC 6.3.5.3)	CL2	nd	no change	COG0047F
MA1964	88	20090812	phosphoribosylformylglycinamidine synthase subunit PurS	phosphoribosylformylglycinamidine synthetase PurS	CL2	Yes	no change	COG1828F
MA1967	262	20090815	CODH nickel-insertion accessory protein	Carbon monoxide dehydrogenase accessory protein cooC	CL2	Yes	no change	COG3640D
MA1979	229	20090827	DNA repair protein RadC	DNA repair protein RadC	CL3	nd	no change	COG2003L
MA1997	112	20090845	hypothetical protein MA1997	Hypothetical protein	CL5	nd	no change	COG0840NT
MA1998	120	20090846	hypothetical protein MA1998	Hypothetical protein	CL5	Yes	no change	-

(a) based on Allen, MA et al., 2009, ISME J. 3: 1012-1035 unless indicated otherwise.

(b) based on Ferguson, JT *et al*, 2009, J. Am. Soc. Mass Spect. 20: 1743-1750; Li, L *et al*, J. Prot Res. 2007 6: 759-771; Rohlin L & Gunsalus RP. 2010 BMC Microbiol. 10:62.

Locus_tag	Length	Gi	Old Product Name	New Product Name (a)	Confidence Level	Expressed in MA (b)	Change in Annotation	COG(s)
MA2008	350	20090856	sodium/calcium exchanger protein	potassium-dependent sodium-calcium exchanger	CL3	nd	no change	COG0530P
MA2016	383	20090864	hypothetical protein MA2016	hypothetical protein	CL5	nd	no change	COG3385L
MA2085	411	20090930	cation efflux system protein	Cation efflux family protein	CL3	Yes	no change	COG0053P, COG1433S
MA2110	267	20090954	multidrug ABC transporter, ATP-binding protein	ABC transporter, ATPase subunit	CL3	Yes	no change	COG1131V
MA2111	294	20090955	heat shock protein HtpX	Peptidase, M48 family	CL4	Yes	more specific	COG0501O
MA2117	112	20090960	hypothetical protein MA2117	Divergent AAA domain protein	CL4	nd	more specific	-
MA2122	917	20090965	deoxyribonuclease	restriction endonuclease	CL4	nd	less specific	COG4096V
MA2142	394	20090985	argininosuccinate synthase	6.3.4.5) (Citrulline-aspartate ligase)	CL2	Yes	no change	COG0137E
MA2143	1070	20090986	carbamoyl phosphate synthase large subunit	Carbamoyl-phosphate synthase large chain (carB) (EC 6.3.5.5)	CL2	Yes	no change	COG0458EF
MA2144	368	20090987	carbamoyl phosphate synthase small subunit	Carbamoyl-phosphate synthase small chain (carA) (EC 6.3.5.5)	CL2	Yes	no change	COG0505EF
MA2145	491	20090988	ABC transporter, ATP-binding protein	ATP-binding protein opuAA (EC 3.6.3.32)	CL2	Yes	no change	COG4175E
MA2146	276	20090989	proW glycine betaine/L-proline ABC transporter, permease	Glycine betaine ABC transporter permease protein opuAB	CL2	nd	no change	COG4176E
MA2147	315	20090990	ABC transporter, solute-binding protein	Glycine betaine ABC transporter substrate binding protein opuAC	CL2	Yes	no change	COG2113E
MA2183	241	20091025	glucose-1-phosphate thymidylyltransferase (rbfA)	Glucose-1-phosphate thymidylyltransferase (dTDP-glucose synthase) (EC:2.7.7.24)	CL2	nd	no change	COG1209M
MA2186	321	20091027	dTDP-glucose 4,6-dehydratase	dTDP-glucose 4,6-dehydratase	CL2	nd	no change	COG1088M
MA2218	136	20091059	potassium channel protein	Hypothetical protein	CL5	Yes	less specific	COG1226P
MA2225	86	20091065	hypothetical protein MA2225	Hypothetical protein	CL5	nd	no change	-
MA2238	282	20091078	hypothetical protein MA2238	protein of unknown function UCP004929	CL4	Yes	more specific	COG4022S
MA2246	426	20091086	squamous cell carcinoma antigen	Serpin (serine protease inhibitor)	CL3	nd	more specific	COG4826O
MA2253	379	20091092	hypothetical protein MA2253	Myo-inositol-1-phosphate synthase (EC 5.5.1.4)	CL2	nd	more specific	COG1260I
MA2266	680	20091104	sensory transduction histidine kinase	Multisensor signal transduction histidine kinase	CL4	Yes	no change	COG0642T, COG2202T, COG3920T
MA2270	166	20091108	hypothetical protein MA2270	Hypothetical protein	CL5	nd	no change	COG3431S
MA2272	455	20091110	protein	DUF214	CL4	nd	less specific	COG0577V
MA2273	395	20091111	hypothetical protein MA2273	hypothetical protein	CL5	Yes	no change	COG1361M
MA2275	237	20091113	protein	ABC transporter, ATPase subunit	CL2	Yes	no change	COG1136V

MA2280	274	20091118	molybdenum ABC transporter, solute-binding protein	molybdate-binding protein (modA)	CL3	nd	no change	COG0725P
MA2286	430	20091124	amino acid transporter	Amino acid transporter domain protein	CL4	nd	no change	COG0531E
MA2289	99	20091127	decarboxylase	(mdrA)	CL3	Yes	more specific	COG0599S
MA2318	77	20091154	hypothetical protein MA2318	Protein with DUF1328 domain	CL4	nd	more specific	-
MA2387	144	20091218	hypothetical protein MA2387	Protein of unknown function (DUF1699)	CL4	nd	more specific	-
MA2388	133	20091219	hypothetical protein MA2388	Hypothetical protein	CL5	nd	no change	-
MA2405	80	20091236	hypothetical protein MA2405	Hypothetical protein	CL5	nd	no change	-
MA2407	296	20091238	pyruvate synthase, beta subunit (porB)	(pyruvate:ferredoxin oxidoreductase beta subunit) (EC 1.2.7.1)	CL2	nd	no change	COG1013C
MA2424	213	20091255	dimethylamine corrinoid protein	dimethylamine corrinoid protein (mtbC)	CL2	nd	no change	COG5012R
MA2429	227	20091260	hypothetical protein MA2429	Hypothetical protein	CL5	nd	no change	-
MA2431	1058	20091262	isoleucyl-tRNA synthetase	Isoleucyl-tRNA synthetase (EC 6.1.1.5)	CL2	Yes	no change	COG0060J
MA2451	271	20091282	hypothetical protein MA2451	type I restriction endonuclease	CL3	nd	more specific	COG0610V
MA2452	368	20091283	hypothetical protein MA2452	Hypothetical protein	CL5	nd	no change	-
MA2498	196	20091329	fumarate hydratase (fumB)	Tartrate dehydratase / Fumarate hydratase, beta subunit	CL3	Yes	more specific	COG1838C
MA2508	174	20091339	hypothetical protein MA2508	DUF367	CL4	Yes	more specific	COG2042S
MA2510	752	20091341	DNA topoisomerase I (topA)	DNA topoisomerase	CL3	Yes	no change	COG0550L, COG0551L
MA2518	307	20091346	hypothetical protein MA2518	RIO2-type Serine Protein Kinase	CL3	nd	more specific	COG0478T
MA2533	404	20091361	nicotinate phosphoribosyltransferase	Nicotinate phosphoribosyltransferase (pncB) (EC 2.4.2.11)	CL2	nd	no change	COG1488H
MA2534	79	20091362	hypothetical protein MA2534	hypothetical protein	CL5	nd	no change	-
MA2548	312	20091375	protein	ABC transporter, ATPase subunit	CL2	nd	no change	COG1136V
MA2557	69	20091384	hypothetical protein MA2557	hypothetical protein	CL5	nd	no change	-
MA2561	330	20091388	hypothetical protein MA2561	HATPase domain-containing multisensor signal transduction histidine kinase	CL4	nd	more specific	COG2202T, COG2203T
MA2584	896	20091410	hypothetical protein MA2584	Hypothetical protein	CL5	nd	no change	-
MA2612	79	20091438	serine proteinase inhibitor	serpin	CL3	nd	more specific	-
MA2628	659	20091451	glycogen debranching enzyme	Hypothetical protein	CL5	Yes	less specific	COG3408G
MA2656	345	20091479	hypothetical protein MA2656	protein of unknown function with UPF0228 domain	CL5	nd	more specific	-
MA2665	491	20091488	phosphomannomutase (pmm)	Phosphoglucomutase/Phosphomannomutase	CL2	nd	more specific	COG1109G

MA2667	804	20091490	phosphoenolpyruvate synthase (ppsA)	Phosphoenolpyruvate synthase, PEP synthase	CL2	nd	no change	COG0574G
MA2701	314	20091525	transposase	Transposase	CL3	nd	no change	COG3039L
MA2715	441	20091539	O-acetylhomoserine (thiol)-lyase	O-acetylhomoserine aminocarboxypropyltransferase (cysD) (EC 2.5.1.49)	CL2	Yes	more specific	COG2873E
MA2748	485	20091571	transposase	transposase	CL4	nd	no change	COG3436L
MA2837	469	20091661	sodium/alanine symporter	Sodium/alanine symporter	CL3	nd	no change	COG1115E
MA2842	351	20091666	methanol dehydrogenase regulatory protein	MoxR-like AAA-3 family ATPase	CL3	nd	more specific	COG0714R
MA2851	260	20091675	synthetase	synthetase	CL1	Yes	no change	COG1635H
MA2862	633	20091686	glutamyl-tRNA(Gln) amidotransferase subunit E	glutamyl-tRNA (Gln) amidotransferase subunit E (EC 6.3.5.7)	CL2	Yes	no change	COG2511J
MA2867	489	20091691	polyferredoxin	4Fe-4S ferredoxin, iron-sulfur binding domain protein	CL4	Yes	more specific	COG1145C
MA2870	93	20091694	hypothetical protein MA2870	Ferredoxin thioredoxin reductase-related protein	CL4	Yes	more specific	COG4802C
MA2896	635	20091717	threonyl-tRNA synthetase (thrS)	Threonyl-tRNA synthetase (EC 6.1.1.3)	CL3	Yes	no change	COG0441J
MA2909	482	20091730	2-oxoisovalerate ferredoxin oxidoreductase, alpha subunit	gamma subunit-like N-terminal region and pyruvate synthase beta subunit-like C-terminal region	CL4	Yes	less specific	COG1013C, COG1014C
MA2910	351	20091731	2-ketoisovalerate ferredoxin reductase	Pyruvate flavodoxin/ferredoxin oxidoreductase-like protein	CL4	Yes	less specific	COG0674C
MA2912	560	20091733	AMP-binding protein	(Acetate--CoA ligase) (EC 6.2.1.1)	CL3	Yes	more specific	COG0365I
MA2914	184	20091735	transcriptional regulator	Protein with helix-turn-helix (HTH) and cupin domain	CL4	Yes	more specific	COG1396K
MA2935	342	20091754	phosphate permease	Inorganic phosphate transporter	CL3	Yes	more specific	COG0306P
MA2947	90	20091766	hypothetical protein MA2947	Hypothetical protein	CL5	nd	no change	-
MA2965	478	20091783	transposase	Hypothetical protein	CL5	nd	less specific	COG5421L
MA2968	484	20091786	hypothetical protein MA2968	Protoporphyrinogen oxidase (EC 1.3.3.4)	CL3	nd	more specific	COG1233Q
MA2969	120	20091787	hypothetical protein MA2969	Hypothetical protein	CL5	nd	no change	COG2146PR
MA2971	217	20091789	monomethylamine corrinoid protein	Monomethylamine corrinoid protein (mtmC)	CL2	Yes	no change	COG5012R
MA2975	282	20091793	hypothetical protein MA2975	Hypothetical protein	CL5	nd	no change	COG0148G
MA2981	136	20091799	hypothetical protein MA2981	Hypothetical protein	CL5	nd	no change	-
MA2987	560	20091805	anthranilate synthase component I	Anthranilate synthase component 1 (trpE) (EC 4.1.3.27)	CL2	Yes	no change	COG0147EH

MA2991	403	20091809	tryptophan synthase subunit beta	Tryptophan synthase beta chain 1 (trpB1) (EC 4.2.1.20)	CL2	Yes	no change	COG0133E
MA3006	171	20091824	formaldehyde-activating enzyme	enzyme, tetrahydromethanopterin-	CL2	Yes	more specific	COG1795S
MA3018	189	20091836	hypothetical protein MA3018	hypothetical protein	CL5	Yes	no change	COG3390S
MA3023	618	20091841	glucosamine-fructose-6-phosphate aminotransferase (isomerizing)	synthetase, contains amidotransferase and phosphosugar isomerase domains	CL2	Yes	no change	COG0449M
MA3024	437	20091842	phosphomannomutase (pmmB)	Phosphoglucomutase/Phosphomannomutase	CL2	Yes	less specific	COG1109G
MA3033	255	20091851	uroporphyrin-III C-methyltransferase	Uroporphyrinogen-III C-methyltransferase (SUMT) (cysGA) (EC 2.1.1.107)	CL2	Yes	no change	COG0007H
MA3052	869	20091870	valyl-tRNA synthetase (valS)	6.1.1.9)	CL3	Yes	no change	COG0525J
MA3055	543	20091873	flagellar assembly protein J	type II secretion system protein F domain	CL3	Yes	more specific	COG1955NU
MA3056	617	20091874	flagella accessory protein I	type II secretion system protein E	CL3	nd	more specific	COG0630NU
MA3057	238	20091875	flagellar accessory protein FlaH	DNA repair protein RadA-domain containing protein	CL4	nd	less specific	COG2874NU
MA3060	236	20091878	hypothetical protein MA3060	hypothetical protein MA3060	CL5	nd	no change	-
MA3061	190	20091879	flagellin	flagellin	CL2	nd	no change	COG1681N
MA3062	215	20091880	flagellin	flagellin	CL2	nd	no change	COG1681N
MA3063	270	20091881	cheR methyltransferase	CheR methyltransferase-like chemotaxis protein	CL3	nd	less specific	COG1352NT
MA3064	220	20091882	chemoreceptor glutamine deamidase CheD	Chemoreceptor glutamine deamidase CheD	CL2	nd	no change	COG1871NT
MA3066	882	20091884	chemotaxis sensor histidine kinase	Chemotaxis protein CheA	CL2	nd	no change	COG0643NT
MA3067	349	20091885	chemotaxis-specific methylesterase	Chemotaxis response regulator protein-glutamate methylesterase CheB	CL2	Yes	more specific	COG2201NT
MA3068	120	20091886	chemotaxis response regulator	Chemotaxis (CheY) protein	CL2	nd	no change	COG0784T
MA3069	266	20091887	hypothetical protein MA3069	DUF439	CL4	nd	more specific	COG2469S
MA3070	182	20091888	chemotaxis signal transduction coupling protein	Chemotaxis protein CheW	CL2	nd	no change	COG0835NT
MA3073	430	20091891	hydroxymethylglutaryl-CoA reductase (NADPH)	3-hydroxy-3-methylglutaryl-CoA reductase (EC 1.1.1.34)	CL2	Yes	no change	COG1257I
MA3085	424	20091903	3-isopropylmalate dehydratase, large subunit	Homoaconitase large subunit (EC 4.2.1.36) (homoaconitate hydratase) (hacA)	CL2	Yes	more specific	COG0065E
MA3118	344	20091936	surface antigen gene	NHL repeat domain (Beta propeller clan) protein	CL4	nd	more specific	COG3391S

MA3120	487	20091938	surface antigen gene	NHL repeat domain (Beta propeller clan) protein	CL4	nd	more specific	COG3391S
MA3121	405	20091939	hypothetical protein MA3121	Hypothetical protein with PepSY-like domain (possible protease inhibitory function)	CL4	nd	more specific	COG2895P
MA3122	445	20091940	surface antigen gene	NHL repeat domain (Beta propeller clan) protein	CL4	nd	more specific	COG3391S
MA3140	392	20091958	mannose-1-phosphate guanylyltransferase (GDP)	Mannose-1-phosphate guanyltransferase (EC 2.7.7.13)	CL3	Yes	no change	COG1208MJ
MA3148	324	20091966	methanol dehydrogenase regulatory protein	MoxR-like AAA-3 family ATPase	CL3	Yes	more specific	COG0714R
MA3152	115	20091970	decarboxylase	(mdrA)	CL2	Yes	more specific	COG0599S
MA3154	287	20091972	hypothetical protein MA3154	Protein of unknown function DUF1130	CL4	nd	more specific	COG5483S
MA3162	117	20091980	hypothetical protein MA3162	Hypothetical zinc-binding protein	CL4	nd	more specific	COG5561S
MA3163	112	20091981	hypothetical protein MA3163	Cupin-domain protein	CL4	nd	more specific	COG0662G
MA3165	242	20091983	hypothetical protein MA3165	Nitrite/sulphite reductase-related protein	CL4	Yes	more specific	COG1251C
MA3166	134	20091984	hypothetical protein MA3166	transcriptional regulator, HxlR family	CL4	nd	more specific	COG1733K
MA3171	274	20091989	protein	ABC transporter, ATPase subunit	CL2	nd	no change	COG1136V
MA3172	460	20091990	hypothetical protein MA3172	Protein of unknown function UCP019164	CL4	nd	more specific	COG1361M
MA3193	484	20092009	amidophosphoribosyltransferase	Amidophosphoribosyltransferase precursor (Glutamine phosphoribosylpyrophosphate amidotransferase) (purF) (EC 2.4.2.14)	CL2	Yes	more specific	COG0034F
MA3194	56	20092010	50S ribosomal protein L37e	(rpl37e)	CL2	nd	no change	COG2126J
MA3195	72	20092011	small nuclear ribonucleoprotein	binding	CL2	Yes	more specific	COG1958K
MA3198	442	20092014	tryptophan synthase subunit beta	Tryptophan synthase beta chain 2 (trpB2) (EC 4.2.1.20)	CL2	Yes	no change	COG1350R
MA3200	137	20092016	hypothetical protein MA3200	Protein of unknown function (DUF1699)	CL4	nd	more specific	-
MA3203	730	20092019	ski2-like helicase	protein	CL3	nd	no change	COG1204R
MA3204	588	20092020	putative ATPase RIL	RNase L inhibitor	CL2	Yes	more specific	COG1245R
MA3211	135	20092027	hypothetical protein MA3211	Protein of unknown function (DUF1699)	CL4	Yes	more specific	-
MA3235	92	20092051	hypothetical protein MA3235	ACT domain-containing protein	CL4	nd	more specific	COG3830T
MA3241	177	20092057	tRNA 2'-O-methylase	tRNA 2'-O-methylase	CL3	nd	no change	COG1303S
MA3242	506	20092058	thymidine phosphorylase	AMP to Ribose-1,5-bisphosphate)	CL2	Yes	more specific	COG0213F

MA3250	485	20092066	cobyric acid synthase	Cobyric acid synthase (cbiP) (EC 6.3.5.10)	CL3	Yes	no change	COG1492H
MA3257	338	20092073	hypothetical protein MA3257	Hypothetical protein with PepSY-like domain (possible protease inhibitory function)	CL4	nd	more specific	-
MA3269	115	20092085	peptidyl-tRNA hydrolase	Peptidyl-tRNA hydrolase (EC 3.1.1.29)	CL2	Yes	no change	COG1990S
MA3279	534	20092094	CTP synthetase	CTP synthase (UTP--ammonia ligase) (pyrG) (EC 6.3.4.2)	CL2	Yes	no change	COG0504F
MA3281	60	20092096	hypothetical protein MA3281	DUF343	CL4	nd	more specific	COG2835S
MA3288	451	20092103	PmbA/TldD family protein	peptidase U62 (DNA gyrase modulator) family protein	CL3	Yes	no change	COG0312R
MA3303	73	20092118	hypothetical protein MA3303	Hypothetical protein	CL5	Yes	no change	-
MA3309	441	20092124	phosphoribosylamine-glycine ligase	ligase (GARS) (purD) (EC 6.3.4.13)	CL2	Yes	no change	COG0151F
MA3310	302	20092125	ornithine carbamoyltransferase	Ornithine carbamoyltransferase (EC 2.1.3.3)	CL2	Yes	no change	COG0078E
MA3317	380	20092131	arylsulfatase regulator	protein of unknown function UCP021940	CL4	Yes*	less specific	COG0641R
MA3322	200	20092136	hypothetical protein MA3322	FMN-binding protein	CL4	Yes	more specific	COG1853R
MA3337	190	20092151	hypothetical protein MA3337	Hypothetical protein	CL5	nd	no change	-
MA3342	405	20092156	trans-homoaconitate synthase (akaS)	(R)-citramalate synthase (cimA) (EC 2.3.3.-)	CL2	Yes	more specific	COG0119E
MA3345	335	20092159	glyceraldehyde-3-phosphate dehydrogenase	glyceraldehyde-3-phosphate dehydrogenase	CL2	Yes	no change	COG0057G
MA3361	370	20092175	transposase	transposase	CL4	Yes	no change	COG0675L
MA3368	1447	20092182	sensory transduction histidine kinase	Multisensor signal transduction histidine kinase	CL4	Yes	no change	COG0642T, COG2202T
MA3373	553	20092187	dihydroxy-acid dehydratase (ilvD)	Dihydroxyacid dehydratase, EC 4.2.1.9	CL2	Yes	no change	COG0129EG
MA3378	96	20092192	hypothetical protein MA3378	hypothetical protein	CL5	Yes	no change	COG0640K
MA3384	73	20092198	hypothetical protein MA3384	PepSY domain protein	CL4	nd	more specific	-
MA3388	424	20092202	serine protease inhibitor	serpin	CL3	nd	more specific	COG4826O
MA3404	254	20092216	GTPase	GTP-binding protein	CL3	nd	less specific	COG1161R
MA3408	802	20092220	phosphoenolpyruvate synthase	Phosphoenolpyruvate synthase, PEP synthase	CL2	Yes	no change	COG0574G
MA3416	203	161484933	30S ribosomal protein S3Ae	SSU ribosomal protein S3Ae (rps3Ae)	CL2	Yes	no change	COG1890J
MA3451	424	20092263	iron ABC transporter, solute-binding protein	transporter substrate binding protein	CL3	Yes	no change	COG0614P
MA3452	377	20092264	iron ABC transporter, permease	Iron complex/Vitamin B12 ABC transporter permease protein	CL3	Yes	no change	COG0609P

MA3453	292	20092265	protein	ABC transporter, ATPase subunit	CL2	nd	more specific	COG1120PH
MA3468	160	20092281	hypothetical protein MA3468	iron dependent repressor	CL3	nd	more specific	COG1321K
MA3496	183	20092306	pyruvoyl-dependent arginine decarboxylase	Pyruvoyl-dependent arginine decarboxylase (EC 4.1.1.19)	CL3	Yes	no change	COG1945S
MA3516	444	20092324	dihydropteroate synthase	dihydropteroate synthase (DHPS) (folP) (EC 2.5.1.15)	CL3	Yes	no change	COG0294H
MA3519	290	20092327	methylenetetrahydrofolate dehydrogenase (NADP+)/methenyltetrahydrofolate cyclohydrolase	(Includes: Methylenetetrahydrofolate dehydrogenase (EC 1.5.1.5); Methenyltetrahydrofolate cyclohydrolase (EC 3.5.4.9))	CL2	Yes	no change	COG0190H
MA3520	412	20092328	glycine hydroxymethyltransferase	Serine hydroxymethyltransferase (EC 2.1.2.1)	CL2	Yes	more specific	COG0112E
MA3522	216	20092330	phosphoribosylglycinamide formyltransferase	Phosphoribosylglycinamide formyltransferase (purN) (EC 2.1.2.2)	CL2	Yes	no change	COG0299F
MA3524	328	20092332	hypothetical protein MA3524	HTH DNA-binding domain protein	CL4	nd	more specific	COG1395K
MA3527	786	20092335	cell division control protein 48 AAA family protein (cdc48)	VCP-like ATPase (VAT), AAA family	CL2	Yes	more specific	COG0464O
MA3529	485	20092337	transposase	transposase	CL4	nd	no change	COG3436L
MA3545	325	20092352	DNA repair and recombination protein RadA	DNA repair and recombination protein radA	CL2	Yes	no change	COG0468L
MA3547	110	20092354	hypothetical protein MA3547	Hypothetical protein	CL5	Yes	no change	-
MA3551	453	20092358	cobalt ABC transporter, ATP-binding protein	Cobalt ABC transporter ATP-binding protein cbiO	CL3	Yes	no change	COG1122P
MA3554	235	20092361	cobalt transport protein CbiM	protein involved in cobalamin biosynthesis, cbiM	CL2	nd	no change	COG0310P
MA3564	395	20092370	acetyltransferase/N-acetylglutamate synthase protein	(EC 2.3.1.35)	CL2	Yes	no change	COG1364E
MA3566	336	20092372	N-acetyl-gamma-glutamyl-phosphate reductase (argJ)	N-acetyl-gamma-glutamyl-phosphate reductase (EC 1.2.1.38) (N-acetyl-glutamate semialdehyde dehydrogenase)	CL2	nd	no change	COG0002E
MA3576	365	20092380	hypothetical protein MA3576	Small-conductance mechanosensitive ion channel	CL3	Yes	more specific	-
MA3582	230	20092386	protein	ABC transporter, ATPase subunit	CL2	nd	no change	COG1136V
MA3583	402	20092387	protein	DUF214	CL4	nd	less specific	COG0577V
MA3584	409	20092388	hypothetical protein MA3584	Hypothetical protein	CL5	Yes	no change	COG1361M
MA3586	214	20092389	TetR family transcriptional regulator	Hypothetical protein	CL5	Yes	less specific	COG1309K
MA3592	416	20092393	phosphoglycerate kinase	2.7.2.3	CL2	Yes	no change	COG0126G
MA3601	209	20092402	hypothetical protein MA3601	Hypothetical protein	CL5	nd	no change	COG2512S

MA3619	94	20092419	MC1	Chromosomal protein MC1	CL2	nd	no change	-
MA3627	265	20092427	nitrogenase (iron protein)	Nitrogenase iron protein (EC:1.18.6.1)	CL2	nd	no change	COG1348P
MA3628	370	20092428	nitrogenase-related protein	Protein containing oxidoreductase/nitrogenase, component 1-like domain	CL4	Yes	more specific	COG2710C
MA3631	130	20092431	sirohydrochlorin cobaltochelatase	cobaltochelatase (CbiXS) (EC 4.99.1.3)	CL2	Yes	no change	COG2138S
MA3665	717	20092465	hypothetical protein MA3665	Hypothetical protein	CL5	nd	no change	COG1203R
MA3670	321	20092470	hypothetical protein MA3670	CRISPR-associated protein Cas1 containing DUF48 domain	CL4	nd	more specific	COG1518L
MA3680	342	20092480	sulfate adenylyltransferase (ADP)	UDP-galactose/glucose pyrophosphorylase (EC 2.7.7.-)	CL3	nd	more specific	COG1085C
MA3686	356	20092486	NAD(P)-dependent glycerol-1-phosphate dehydrogenase	glycerol-1-phosphate dehydrogenase (EC 1.1.1.261)	CL2	Yes	no change	COG0371C
MA3689	387	20092489	5-formaminoimidazole-4-carboxamide-1-(beta)-D-ribofuranosyl 5'-monophosphate synthetase-like protein	5-formaminoimidazole-4-carboxamide-1-(beta)-D-ribofuranosyl 5'-monophosphate synthetase (EC 6.3.4.-) (5-aminoimidazole-4-carboxamide-1-beta-D-ribofuranosyl 5'-monophosphate--formate ligase) (purP)	CL2	Yes	more specific	COG1759R
MA3690	443	20092490	translation initiation factor IF-2 subunit gamma	translation initiation factor a/eIF-2 gamma subunit	CL2	Yes	no change	COG5257J
MA3692	194	20092492	DNA-directed RNA polymerase subunit E'	DNA-directed RNA polymerase subunit E', rpoE1	CL2	Yes	no change	COG1095K
MA3696	54	20092496	30S ribosomal protein S27ae	SSU ribosomal protein S27AE (rps27ae)	CL2	nd	no change	COG1998J
MA3705	547	20092505	O-sialoglycoprotein endopeptidase/protein kinase	with atypical AP endonuclease activity	CL2	Yes	more specific	COG0533O, COG3642T
MA3706	184	20092506	Ham1 protein	Nucleotide-triphosphatase (EC 3.6.1.-)	CL2	Yes	more specific	COG0127F
MA3710	68	20092509	hypothetical protein MA3710	hypothetical protein MA3710	CL5	nd	no change	COG1145C
MA3720	370	20092518	transposase	transposase	CL4	nd	no change	COG0675L
MA3722	89	20092519	hypothetical protein MA3722	Hypothetical protein	CL5	nd	no change	-
MA3732	346	20092529	F420H2 dehydrogenase subunit F	F420H2 dehydrogenase subunit F (fpoF)	CL2	Yes	no change	COG1035C

MA3733	328	20092530	methylenetetrahydromethanopterin reductase	Coenzyme F420-dependent N(5),N(10)-methylenetetrahydromethanopterin reductase (mer) (EC 1.5.99.11)	CL2	Yes	no change	COG2141C
MA3734	76	20092532	hypothetical protein MA3734	Hydantoinase/oxoprolinase	CL3	Yes	more specific	COG4855S
MA3736	117	20092534	carboxymuconolactone decarboxylase	protein disulfide reductase (mdrA)	CL1	Yes	more specific	COG0599S
MA3740	221	20092538	iron-sulfur flavoprotein	reductase	CL3	Yes	more specific	COG0655R
MA3741	207	20092539	flavoprotein	Protein with flavoprotein domain	CL4	Yes	less specific	COG1036C
MA3751	166	20092549	3-isopropylmalate dehydratase	3-Isopropylmalate dehydratase small subunit (3-isopropylmalate isomerase small subunit) and homoaconitase small subunit - dual specificity	CL2	Yes	more specific	COG0066E
MA3757	351	20092555	mannosyltransferase B	hypothetical protein	CL5	nd	less specific	COG0438M
MA3777	238	20092573	glucose-1-phosphate thymidylyltransferase	Glucose-1-phosphate thymidylyltransferase (dTDP-glucose synthase) (EC:2.7.7.24)	CL2	Yes	no change	COG1209M
MA3778	269	20092574	dTDP-4-dehydrorhamnose reductase	dTDP-4-dehydrorhamnose reductase (EC:1.1.1.133)	CL2	Yes	no change	COG1091M
MA3779	318	20092575	dTDP-glucose 4,6-dehydratase	dTDP-glucose 4,6-dehydratase	CL2	nd	no change	COG1088M
MA3780	183	20092576	dTDP-4-dehydrorhamnose 3,5-epimerase	dTDP-4-dehydrorhamnose 3,5-epimerase related protein	CL3	Yes	no change	COG1898M
MA3781	460	20092577	mannose-1-phosphate guanylyltransferase (GDP)	Xanthan-like biosynthesis protein (Includes: Mannose-6-phosphate isomerase (Phosphomannose isomerase) (PMI) (Phosphohexomutase); Mannose-1-phosphate guanylyl transferase (GDP) (GDP-mannose pyrophosphorylase) (GMP))	CL2	Yes	more specific	COG0662G, COG0836M
MA3787	469	20092583	putative oxidoreductase	(flavoprotein) subunit SudA (EC 1.97.-.-)	CL2	Yes	more specific	COG0493ER
MA3790	335	20092586	ketol-acid reductoisomerase	Ketol-acid reductoisomerase (EC 1.1.1.86) (Acetohydroxy-acid isomeroreductase)	CL2	Yes	no change	COG0059EH
MA3791	161	20092587	acetolactate synthase 3 regulatory subunit	acetolactate synthase, small subunit (EC 2.2.1.6)	CL2	Yes	no change	COG0440E

MA3792	564	20092588	acetolactate synthase 3 catalytic subunit	acetolactate synthase, large subunit (acetohydroxyacid synthase) (EC 2.2.1.6)	CL2	Yes	no change	COG0028EH
MA3793	483	20092589	(R)-citramalate synthase	(R)-citramalate synthase (cimA) (EC 2.3.3.-)	CL2	Yes	no change	COG0119E
MA3800	413	20092596	transposase	Hypothetical protein	CL5	nd	less specific	COG5421L
MA3840	362	20092636	hypothetical protein MA3840	Hypothetical protein	CL5	nd	no change	COG4748S
MA3845	126	20092641	hypothetical protein MA3845	Hypothetical protein	CL5	nd	no change	-
MA3849	348	20092645	cellulase	Archaeal aminopeptidase or endoglucanase M (cellulase M)	CL2	Yes	more specific	COG1363G
MA3850	117	20092646	prefoldin, subunit beta	Prefoldin beta subunit (GimC beta subunit) (pfdB)	CL2	Yes	no change	COG1382O
MA3851	334	20092647	phosphotransferase	RecJ-like protein	CL4	nd	less specific	COG0618R
MA3852	485	20092648	transposase	transposase	CL4	Yes	no change	COG3436L
MA3853	434	20092649	coenzyme F390 synthetase	Phenylacetate-coenzyme A ligase (EC 6.2.1.30)	CL2	Yes	more specific	COG1541H
MA3854	145	20092650	subunit	protein	CL4	Yes	less specific	COG4747R
MA3857	195	20092653	hypothetical protein MA3857	DUF88	CL4	Yes	more specific	COG1432S
MA3860	805	20092656	acetyl-CoA decarbonylase/synthase complex subunit alpha	Acetyl-CoA decarbonylase/synthase complex alpha subunit 2 (EC 1.2.99.2)	CL2	Yes	no change	COG1152C
MA3861	170	20092657	carbon-monoxide dehydrogenase accessory protein	carbon-monoxide dehydrogenase accessory protein	CL2	Yes	no change	COG3640D
MA3862	470	20092658	decarbonylase/synthase complex subunit beta	decarbonylase/synthase complex beta subunit (EC 2.3.1.-)	CL2	Yes	no change	COG1614C
MA3863	253	20092659	carbon-monoxide dehydrogenase accessory protein	carbon monoxide dehydrogenase/acetyl-CoA synthase complex, nickel-inserting subunit (cooC)	CL3?	Yes	no change	COG3640D
MA3864	436	20092660	decarbonylase/synthase complex subunit delta	decarbonylase/synthase complex delta subunit	CL2	Yes	no change	COG2069C
MA3865	468	20092661	decarbonylase/synthase complex subunit gamma	decarbonylase/synthase complex gamma subunit (EC 2.1.1.-)	CL2	Yes	no change	COG1456C
MA3874	637	20092670	cleavage and polyadenylation specificity factor	RNA-metabolizing metallo-beta-lactamase protein	CL3	Yes	more specific	COG1782R
MA3876	392	20092672	cell division protein FtsZ	Cell division protein FtsZ	CL2	Yes	no change	COG0206D
MA3880	671	20092676	membrane-bound proton-translocating pyrophosphatase	Pyrophosphate-energized proton pump / Pyrophosphate-energized inorganic pyrophosphatase (H(+)-PPase) (EC 3.6.1.1) (hppA)	CL2	nd	no change	COG3808C
MA3884	126	20092680	hypothetical protein MA3884	Protein of unknown function DUF1621	CL4	Yes	more specific	COG2450S

MA3886	480	20092682	prolyl-tRNA synthetase	6.1.1.15)	CL2	Yes	no change	COG0442J
MA3937	350	20092733	hypothetical protein MA3937	permease	CL4	nd	more specific	COG0701R
MA3938	77	20092734	hypothetical protein MA3938	Thioredoxin (EC 1.8.4.8)	CL2	nd	more specific	COG0526OC
MA3942	79	20092738	hypothetical protein MA3942	small redox protein	CL3	nd	more specific	COG0526OC
MA3957	213	20092753	protein	ABC transporter, ATPase subunit	CL2	nd	no change	COG1136V
MA3960	302	20092756	protein	ABC transporter, ATPase subunit	CL2	nd	no change	COG1136V
MA3969	247	20092764	geranylgeranylglyceryl phosphate synthase-like protein	geranylgeranylglyceryl phosphate synthase (EC 2.5.1.42)	CL3	Yes	more specific	COG1646R
MA3971	451	20092766	adenylosuccinate lyase	adenylosuccinate lyase (purB) (EC 4.3.2.2)	CL2	Yes	no change	COG0015F
MA3987	128	20092781	translation initiation factor IF-5A	translation initiation factor aIF-5A	CL2	Yes	no change	COG0231J
MA3994	415	20092789	BadF/BadG/BcrA/BcrD ATPase	2-hydroxyglutaryl-CoA dehydratase component A	CL3	nd	more specific	COG1924I
MA3995	170	20092790	hypothetical protein MA3995	protein of unknown function UCP018781	CL4	Yes	more specific	COG4050S
MA3998	537	20092793	ABC transporter, ATP-binding protein	Methylcoenzyme M reductase system component A2	CL2	Yes	more specific	COG1123R
MA4002	100	20092797	hypothetical protein MA4002	Hypothetical protein	CL5	nd	no change	-
MA4007	521	20092802	phosphoglyceromutase	Cofactor-independent Phosphoglycerate mutase	CL2	Yes	no change	COG0696G
MA4008	201	20092803	Fun34 related protein	protein	CL4	nd	more specific	COG1584S
MA4012	538	20092807	phosphoribosylaminoimidazolecarboxamide formyltransferase	Bifunctional purine biosynthesis protein purH (Includes: Phosphoribosylaminoimidazolecarboxamide formyltransferase (EC 2.1.2.3) (AICAR transformylase); IMP cyclohydrolase (EC 3.5.4.10) (Inosinicase) (IMP synthetase) (ATIC))	CL2	Yes	no change	COG0138F
MA4033	456	20092826	surface antigen gene	NHL repeat domain (Beta propeller clan) protein	CL4	Yes	more specific	COG3391S
MA4036	310	20092829	protein	ABC transporter, ATPase subunit	CL3	nd	more specific	COG1131V
MA4037	250	20092830	hypothetical protein MA4037	hypothetical protein	CL5	nd	no change	COG1277R
MA4038	125	20092831	hypothetical protein MA4038	Hypothetical protein	CL5	nd	no change	-
MA4040	246	20092833	hypothetical protein MA4040	HTH DNA-binding domain protein	CL4	Yes	more specific	COG1709K
MA4041	349	20092834	hypothetical protein MA4041	3-hydroxy-3-methylglutaryl-CoA synthase (EC 4.1.3.5)	CL3	Yes	more specific	COG3425I
MA4042	390	20092835	acetyl-CoA acetyltransferase	Acetoacetyl-CoA thiolase (EC 2.3.1.9)	CL3	Yes	more specific	COG0183I
MA4043	132	20092836	hypothetical protein MA4043	DUF35-domain protein	CL4	Yes	more specific	COG1545R
MA4046	712	20092839	methionyl-tRNA synthetase	Methionyl-tRNA synthetase (6.1.1.10)	CL2	Yes	no change	COG0073R, COG0143J

MA4052	396	20092845	alpha-amylase	Glycoside hydrolase family 57 protein	CL4	Yes	more specific	COG1449G
MA4055	715	20092848	phosphoribosylformylglycinamidine synthase II	Phosphoribosylformylglycinamidine synthase II (FGAM synthase II) (purL) (EC 6.3.5.3)	CL2	nd	no change	COG0046F
MA4059	102	20092852	translation initiation factor Sui1	translation initiation factor SUI1	CL2	Yes	no change	COG0023J
MA4060	121	20092853	hypothetical protein MA4060	Hypothetical protein	CL5	Yes	no change	COG2522R
MA4061	408	20092854	hypothetical protein MA4061	protein of unknown function with DUF650 and DUF651 domains	CL4	nd	more specific	COG1602S
MA4063	262	20092856	phosphoribosylaminoimidazole-succinocarboxamide synthase	Phosphoribosylaminoimidazole-succinocarboxamide synthase (SAICAR synthetase) (purC) (EC 6.3.2.6)	CL2	nd	no change	COG0152F
MA4064	428	20092857	hypothetical protein MA4064	AAA-family ATPase	CL3	nd	more specific	COG0464O
MA4073	74	20092866	hypothetical protein MA4073	Hypothetical protein	CL5	nd	no change	-
MA4075	140	20092868	regulator	Nickel-responsive regulator	CL3	Yes	more specific	COG0864K
MA4080	114	20092873	hypothetical protein MA4080	Hypothetical protein	CL5	Yes	no change	-
MA4093	284	20092886	hypothetical protein MA4093	synthase) domain-containing protein	CL4	Yes	more specific	COG0517R
MA4099	138	20092892	hypothetical protein MA4099	DUF356	CL4	nd	more specific	COG1844S
MA4100	447	20092893	gamma-glutamyl phosphate reductase	dehydrogenase (proA) (EC 1.2.1.41)	CL1	Yes	no change	COG0014E
MA4101	385	20092894	gamma-glutamyl kinase	Glutamate 5-kinase (proB) (EC 2.7.2.11)	CL1	Yes	no change	COG0263E
MA4102	270	20092895	pyrroline-5-carboxylate reductase	Pyrroline-5-carboxylate reductase (proC) (EC 1.5.1.2)	CL1	Yes	no change	COG0345E
MA4103	219	20092896	peroxiredoxin	3-Cys thioredoxin peroxidase (EC 1.11.1.15)	CL2	Yes	more specific	COG0450O
MA4109	417	20092902	signal recognition particle receptor	signal recognition docking protein FtsY	CL2	Yes	more specific	COG0552U
MA4111	61	20092904	50S ribosomal protein LX	LSU ribosomal protein LX (rplX)	CL2	Yes	no change	COG2157J
MA4114	51	20092907	50S ribosomal protein L39e	(rpl39e)	CL2	Yes	no change	COG2167J
MA4116	122	20092909	hypothetical protein MA4116	protein	CL2	Yes	more specific	COG2118R
MA4117	149	20092910	30S ribosomal protein S19e	(rps19e)	CL2	Yes	no change	COG2238J
MA4118	424	20092911	adenylosuccinate synthetase	Adenylosuccinate synthetase (purA) (EC 6.3.4.4)	CL2	Yes	no change	COG0104F
MA4123	340	20092916	proteasome-activating nucleotidase	Proteasome-activating nucleotidase (Proteasome regulatory subunit) (pan)	CL2	Yes	no change	COG1222O
MA4151	402	20092944	protein	Radical SAM family protein	CL4	Yes	more specific	COG0535R
MA4154	82	20092947	H(+)-transporting ATP synthase, subunit C	H(+)-transporting ATP synthase, subunit K (EC 3.6.3.14)	CL2	Yes	more specific	COG0636C

MA4157	101	20092950	V-type ATP synthase subunit F	V-type ATP synthase subunit F (atpF) (EC 3.6.3.14)	CL2	Yes	no change	COG1436C
MA4158	578	20092951	V-type ATP synthase subunit A	V-type ATP synthase alpha chain (atpA) (EC 3.6.3.14)	CL2	Yes	no change	COG1155C
MA4159	460	20092952	V-type ATP synthase subunit B	V-type ATP synthase beta chain (atpB) (EC 3.6.3.14)	CL2	Yes	no change	COG1156C
MA4160	209	20092953	V-type ATP synthase subunit D	V-type ATP synthase subunit D (atpD) (EC 3.6.3.14)	CL2	Yes	no change	COG1394C
MA4169	135	20092962	hypothetical protein MA4169	Protein of unknown function (DUF1699)	CL4	Yes	more specific	-
MA4171	153	20092964	hypothetical protein MA4171	Protein of unknown function (DUF1699)	CL4	nd	more specific	-
MA4174	346	20092967	formylmethanofuran dehydrogenase, subunit F	reductase iron-sulfur subunit A (hdrA) with C-terminal mvhD-like electron transfer domain (EC 1.8.98.1)	CL2	nd	more specific	COG1145C
MA4175	584	20092968	formylmethanofuran dehydrogenase, subunit A	Molybdenum formylmethanofuran dehydrogenase subunit A (fmdA) (EC 1.2.99.5)	CL2	Yes	more specific	COG1229C
MA4177	129	20092970	formylmethanofuran dehydrogenase, subunit D	Molybdenum formylmethanofuran dehydrogenase subunit D (fmdD) (EC 1.2.99.5)	CL2	Yes	more specific	COG1153C
MA4178	433	20092971	formylmethanofuran dehydrogenase, subunit B	Molybdenum formylmethanofuran dehydrogenase subunit B (fmdB) (EC 1.2.99.5)	CL2	nd	more specific	COG1029C
MA4194	58	20092985	ferredoxin	4Fe-4S ferredoxin, iron-sulfur protein	CL4	nd	no change	COG1145C
MA4195	323	20092986	nitrogen fixation protein	FeMo cofactor biosynthesis protein nifB	CL2	Yes	more specific	COG0535R
MA4196	120	20092987	6-pyruvoyltetrahydropterin synthase	6-pyruvoyl tetrahydrobiopterin synthase (PTPS) (EC 4.2.3.12)	CL2	nd	no change	COG0720H
MA4198	186	20092989	hypothetical protein MA4198	protein	CL4	Yes	more specific	COG2029S
MA4226	211	20093016	hypothetical protein MA4226	Hypothetical protein	CL5	nd	no change	-
MA4230	315	20093020	hypothetical protein MA4230	Hypothetical protein	CL5	nd	no change	-
MA4245	112	20093035	HTH DNA Binding protein	DUF134	CL4	Yes	more specific	COG1342R
MA4258	240	20093048	precorrin-8X methylmutase	Cobalt-precorrin-8X methylmutase cbiC (EC 5.4.1.-)	CL3	Yes	no change	COG2082H
MA4259	266	20093049	precorrin-3B C17-methyltransferase	methyltransferase cbiH (EC 2.1.1.-)	CL2	Yes	more specific	COG1010H
MA4261	242	20093050	precorrin-4 C11-methyltransferase	methyltransferase cbiF (EC 2.1.1.-)	CL2	Yes	more specific	COG2875H

MA4262	202	20093051	cobalt-precorrin-2 C(20)-methyltransferase	cobalt-precorrin-2 C(20)-methyltransferase (cbiL)(EC 2.1.1.151)	CL3	nd	no change	COG2243H
MA4264	443	20093053	isopropylmalate/homocitrate/citramalate synthase family protein	Re-Citrate Synthase	CL2	Yes	more specific	COG0119E
MA4265	342	20093054	isocitrate/isopropylmalate dehydrogenase family protein	Isocitrate/isopropylmalate dehydrogenase	CL2	Yes	more specific	COG0473CE
MA4266	280	20093055	hypothetical protein MA4266	TatD-related deoxyribonuclease	CL4	Yes	more specific	COG1831R
MA4271	374	20093060	cell division protein FtsZ	Cell division protein FtsZ	CL2	Yes	no change	COG0206D
MA4273	156	20093062	transcription antitermination protein NusG	LSU ribosomal protein L26e (rpl26e) (NusG)	CL3	Yes	no change	COG0250K
MA4274	161	20093063	50S ribosomal protein L11P	(rpl11p)	CL2	Yes	no change	COG0080J
MA4275	213	20093064	50S ribosomal protein L1P	(rpl1p)	CL2	Yes	no change	COG0081J
MA4277	104	20093066	50S ribosomal protein L12P	(rpl12p)	CL2	Yes	no change	COG2058J
MA4329	428	20093117	thiamine biosynthesis protein ThiC	Thiamine biosynthesis protein thiC (thiC)	CL2	Yes	no change	COG0422H
MA4331	183	20093119	transcription factor	TATA-box binding protein TBP3	CL1	Yes	more specific	COG2101K
MA4349	294	20093137	branched-chain amino acid aminotransferase	Branched-chain amino acid aminotransferase (ilvE) (Transaminase B) (EC 2.6.1.42)	CL2	Yes	no change	COG0115EH
MA4351	364	20093139	GTP-binding protein	domain	CL2	Yes	more specific	COG1163R
MA4378	929	20093165	sodium/potassium-transporting ATPase, alpha subunit	Cation-transporter, P-type ATPase (EC 3.6.3.-)	CL2	Yes	less specific	COG0474P
MA4379	342	20093166	methylcobalamin:coenzyme M methyltransferase	methanol-specific methylcobalamin:CoM methyltransferase (mtaA)	CL1	Yes	more specific	COG0407H
MA4380	539	2E +7	hypothetical protein	corrinoid activation protein (ramM)	CL2	Yes	more specific	
MA4382	451	20093169	CobW protein	nucleotide-binding CobW/HypB/UreG-like protein	CL4	Yes*	less specific	COG0523R
MA4388	83	20093175	hypothetical protein MA4388	Hypothetical protein	CL5	nd	no change	-
MA4391	258	20093178	methanol-5-hydroxybenzimidazolylcobamide co-methyltransferase, isozyme 2	corrinoid-containing methyl-accepting protein (mtaC2)	CL1	Yes*	more specific	COG5012R
MA4392	461	20093179	methanol-5-hydroxybenzimidazolylcobamide co-methyltransferase, isozyme 2	methanol-5-hydroxybenzimidazolylcobamide co-methyltransferase MtaB2 (EC 2.1.1.90)	CL1	Yes	no change	-
MA4399	805	20093186	acetyl-CoA decarbonylase/synthase complex subunit alpha	Acetyl-CoA decarbonylase/synthase complex alpha subunit 2 (EC 1.2.99.2)	CL2	Yes	no change	COG1152C

MA4401	394	20093188	translation-associated GTPase	GTP-binding protein	CL2	Yes	less specific	COG0012J
MA4406	247	20093193	protein	atypical ABC-ATPase SufC	CL2	Yes	more specific	COG0396O
MA4407	407	20093194	hypothetical protein MA4407	sufB	CL4	Yes	more specific	COG0719O
MA4413	543	20093200	Hsp60	(Chaperonin)	CL2	Yes	no change	COG0459O
MA4415	252	20093202	3-isopropylmalate dehydratase	DUF75-domain conserved archaeal protein	CL4	Yes	less specific	COG1938R
MA4425	123	20093211	hypothetical protein MA4425	hypothetical protein	CL5	nd	no change	COG4744S
MA4428	285	20093214	hypothetical protein MA4428	Hypothetical protein	CL5	Yes	no change	COG1340S
MA4430	279	20093216	F420-dependent methylenetetrahydromethanopterin dehydrogenase	F420-dependent methylenetetrahydromethanopterin dehydrogenase (EC 1.5.99.9)	CL2	Yes	no change	COG1927C
MA4438	600	20093224	hypothetical protein MA4438		CL5	Yes	no change	-
MA4448	311	20093234	myo-inositol 2-dehydrogenase	Oxidoreductase (NAD- or NADP-binding)-like protein	CL3	nd	less specific	COG0673R
MA4457	427	20093243	UDP-glucose 6-dehydrogenase	UDP-glucose/GDP-mannose dehydrogenase family protein	CL2	nd	less specific	COG1004M
MA4459	318	20093245	UTP-glucose-1-phosphate uridylyltransferase	UDP-glucose pyrophosphorylase	CL2	Yes	more specific	COG1210M
MA4460	320	20093246	dTDP-glucose 4,6-dehydratase	UDP-galactose -4-epimerase	CL3	Yes	more specific	COG0451MG
MA4472	64	20093257	30S ribosomal protein S17e	(rps17e)	CL2	Yes	no change	COG1383J
MA4474	263	20093259	dihydrodipicolinate reductase	Dihydrodipicolinate reductase (EC 1.3.1.26)	CL2	Yes	no change	COG0289E
MA4485	151	20093270	hypothetical protein MA4485	Hypothetical protein	CL5	nd	no change	-
MA4511	305	20093296	GMP synthase subunit B	hydrolyzing) subunit B (EC 6.3.5.2)	CL2	Yes	no change	COG0519F
MA4515	299	20093300	acetylglutamate kinase	2.7.2.8)	CL2	Yes	no change	COG0548E
MA4516	94	20093301	MC1	Chromosomal protein MC1	CL2	nd	no change	-
MA4517	335	20093302	GTP cyclohydrolase	GTP cyclohydrolase MptA (EC 3.5.4.-)	CL2	Yes	no change	COG1469S
MA4519	477	20093304	hypothetical protein MA4519	protein of unknown function UCP016937	CL4	Yes	more specific	COG4065S
MA4522	93	20093307	aspartyl/glutamyl-tRNA amidotransferase subunit C	Aspartyl/Glutamyl-tRNA (Asn/Gln) amidotransferase subunit C (EC 6.3.5.-)	CL2	Yes	no change	COG0721J
MA4523	476	20093308	glutamyl-tRNA (Gln) amidotransferase, subunit A	glutamyl-tRNA (Gln) amidotransferase subunit A (EC 6.3.5.-)	CL2	Yes	no change	COG0154J
MA4524	495	20093309	aspartyl/glutamyl-tRNA amidotransferase subunit B	Aspartyl/Glutamyl-tRNA (Asn/Gln) amidotransferase subunit B (EC 6.3.5.-)	CL2	Yes	no change	COG0064J
MA4532	258	20093317	iron compounds ABC transporter, ATP-binding protein	ABC transporter, ATPase subunit	CL2	nd	less specific	COG1120PH

MA4542	286	20093326	small heat shock protein	Peptidase, M48 family	CL4	Yes	more specific	COG0501O
MA4543	230	20093327	thymidylate synthase	2.1.1.45)	CL2	Yes	no change	COG0207F
MA4546	570	20093330	methyl coenzyme M reductase, subunit alpha	subunit alpha (mcrA) (EC 2.8.4.1)	CL2	Yes	no change	COG4058H
MA4547	248	20093331	methyl coenzyme M reductase, subunit gamma	subunit gamma (mcrG) (EC 2.8.4.1)	CL2	Yes	no change	COG4057H
MA4548	206	20093332	methyl coenzyme M reductase, subunit D	coenzyme M reductase operon protein C (mcrC) with unknown function	CL2	Yes	less specific	COG4056H
MA4550	434	20093334	methyl coenzyme M reductase, subunit beta	methyl-coenzyme M reductase, subunit beta (mcrB) (EC 2.8.4.1)	CL2	Yes	no change	COG4054H
MA4552	1145	20093336	DNA polymerase II large subunit	DNA polymerase II, large subunit	CL2	Yes	no change	COG1933L
MA4569	518	20093353	putative monovalent cation/H+ antiporter subunit D	Na+/H+ antiporter subunit	CL2	Yes	no change	COG0651CP
MA4572	806	20093356	putative monovalent cation/H+ antiporter subunit A	Na+/H+ antiporter subunit	CL2	Yes	more specific	COG1009CP, COG2111P
MA4575	753	20093359	cell division control protein 48	CDC48, AAA family ATPase	CL3	Yes	more specific	COG0464O
MA4587	440	20093371	Srp54	signal recognition particle SRP54	CL2	Yes	no change	COG0541U
MA4590	189	20093374	GMP synthase subunit A	hydrolyzing) subunit A (EC 6.3.5.2)	CL2	nd	no change	COG0518F
MA4591	265	20093375	fructose-bisphosphate aldolase	2-amino-3,7-dideoxy-D-threo-hept-6-ulosonate synthase CL2	CL2	Yes	more specific	COG1830G
MA4592	380	20093376	3-dehydroquinate synthase	3-dehydroquinate synthase (EC 4.6.1.3)	CL2	Yes	no change	COG1465E
MA4594	280	20093378	shikimate 5-dehydrogenase	Shikimate dehydrogenase (aroE) (EC 1.1.1.25)	CL2	Yes	no change	COG0169E
MA4607	222	20093391	triosephosphate isomerase	Triosephosphate isomerase EC 5.3.1.1	CL2	Yes	no change	COG0149G
MA4608	392	20093392	bifunctional formaldehyde-activating enzyme/3-hexulose-6-phosphate synthase	(Includes: Formaldehyde-activating enzyme (Fae) (EC 4.3.-.-) & 3-hexulose-6-phosphate synthase (HPS) (EC 4.1.2.-)) (fae-hps)	CL2	Yes	no change	COG0269G, COG1795S
MA4615	515	20093396	2-isopropylmalate synthase	2-isopropylmalate synthase (EC 2.3.3.13)	CL2	Yes	no change	COG0119E
MA4618	614	161484932	hypothetical protein MA4618	protein of unknown function UPF0313	CL4	nd	more specific	COG1032C
MA4628	301	20093407	reverse transcriptase	Reverse transcriptase, RNA dependent DNA polymerase	CL4	nd	no change	COG3344L
MA4635	519	20093414	DNA primase	Toprim (topoisomerase-primase) domain protein	CL4	Yes	more specific	COG0358L
MA4642	127	20093421	hypothetical protein MA4642	hypothetical protein	CL5	nd	no change	COG4744S

MA4652	263	20093431	hypothetical protein MA4652	DUF124	CL4	Yes	more specific	COG2013S
MA4657	104	20093436	hypothetical protein MA4657	Hypothetical protein	CL5	nd	no change	-
MA4668	79	20091114	transcriptional regulator	containing transcriptional regulator	CL3	nd	more specific	COG1476K
MA4672	45	20090625	DNA-directed RNA polymerase subunit P	DNA-directed RNA polymerase subunit P, rpoP	CL2	nd	no change	COG1996K
MA4675	512	20091516	transposase	Hypothetical protein	CL5	nd	less specific	COG0675L

(a) based on Allen, MA et al., 2009, ISME J. 3: 1012-1035 unless indicated otherwise.

(b) based on Ferguson, JT et al, 2009, J. Am. Soc. Mass Spect. 20: 1743-1750; Li, L et al, J. Prot Res. 2007 6: 759-771; Rohlin L & Gunsalus RP. 2010 BMC Microbiol. 10:62.
