# Supplementary figures and images for: A nuclear magnetic resonance based approach to accurate functional annotation of putative enzymes in the methanogen Methanosarcina acetivorans
Source: BMC Genomics. 2011 Jun 15;12(Suppl 1):S7. doi: 10.1186/1471-2164-12-S1-S7 (PMC3223730; doi:10.1186/1471-2164-12-S1-S7)

**(a)**

**Change in annotation**

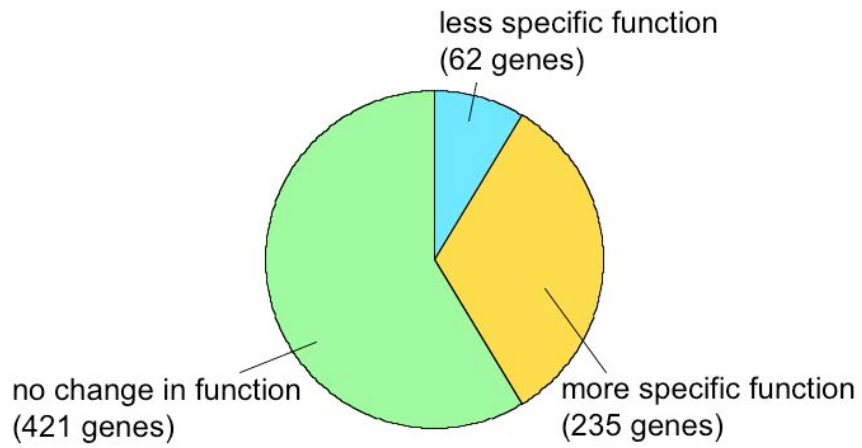

**(b)**

**Distribution of confidence levels (CL)**

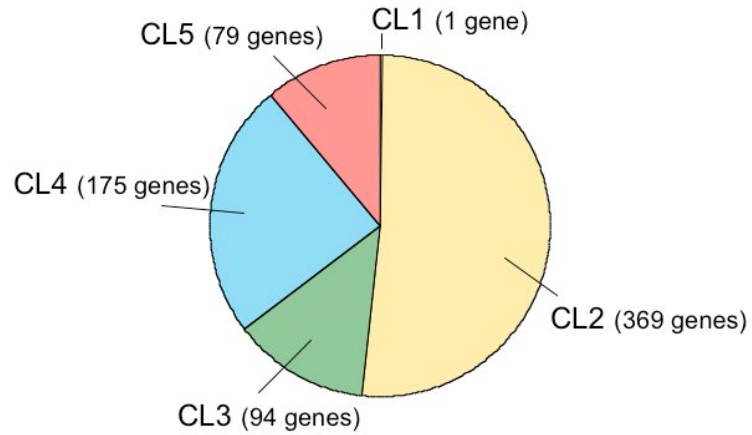

Supplement: Additional file 2 — Summary of results for manually re-annotated MA genes. (a) Categorization of revised MA annotations as more specific, less specific or no change. (b) The distribution of confidence levels (defined in the text) in re-annotated MA genes. [file 1471-2164-12-S1-S7-S2.pdf]
