# Supplementary material for: A nuclear magnetic resonance based approach to accurate functional annotation of putative enzymes in the methanogen Methanosarcina acetivorans
Source: BMC Genomics. 2011 Jun 15;12(Suppl 1):S7. doi: 10.1186/1471-2164-12-S1-S7 (PMC3223730; doi:10.1186/1471-2164-12-S1-S7)
Supplement: Additional file 3 — List of forward and reverse primer DNA sequences used for cloning MA target genes. [file 1471-2164-12-S1-S7-S3.pdf]

Additional Table 2: List of forward and reverse primer DNA sequences used for cloning MA target genes.

GENE	FORWARD	REVERSE
MA0154	5’GCGCGCCATATGATCAAAAAA ATGGCAACCGAG-3'	5'TTATTAGTCGACCTAGCACCTCAG GACAGTTTCGAATTCT-3'
MA0246	5'CACAATGCCATATGACTTATAG AGATTTTATAG-3'	5'TGTGTGGTCGACTTACTCTTCAGG AATCACTGC-3'
MA0940	5'GCATGTCGACTCAGTCTTTCCCC TCAAACTC-3'	5'ATATCATATGACCGTTTCGGAGA AGATC-3'
MA2498	5'CACAATGCCATATGGAGTACCA CCTGCAGAC-3'	5'TGTGTGGTCGACTTACTTCTGCAT TCCTTCATAC-3'
MA3520	5'ATGTATGGATTTAGGTGACACA TATGTCTTAC-3'	5'CAATGATAAACCTCCCGAATTCG CTGTAAACAGCTCGAGTTCGCTGT AAACAGG-3'
MA3706	5'GAGATAAAAAAGAGGGCTCCA TATGC-3'	5'GGGATGCAGGGTTTGAATTCCTT GCTGAGCTCGAGTTCCTTGCTGG-3'
MA4265	5'CACGCTTTATAGATCTGGTGAT CATATGAC-3'	5'CAGATGCTCGAAATCTAAGCTTC AG-3'
